# From NMR to AI: Do We Need ^1^H NMR Experimental Spectra to Obtain High-Quality logD Prediction Models?

**DOI:** 10.1021/acs.jcim.4c02145

**Published:** 2025-03-05

**Authors:** Arkadiusz Leniak, Wojciech Pietruś, Aleksandra Świderska, Rafał Kurczab

**Affiliations:** † Department of Medicinal Chemistry, Celon Pharma S.A., ul. Marymoncka 15, Kazun Nowy 05-152, Poland; ‡ Department of Medicinal Chemistry, Maj Institute of Pharmacology, Polish Academy of Sciences, Smetna 12, Krakow 31-343, Poland; § Faculty of Mathematics and Natural Sciences, Department of Chemistry, University of Applied Sciences in Tarnow, Mickiewicza 8, Tarnow 33-100, Poland

## Abstract

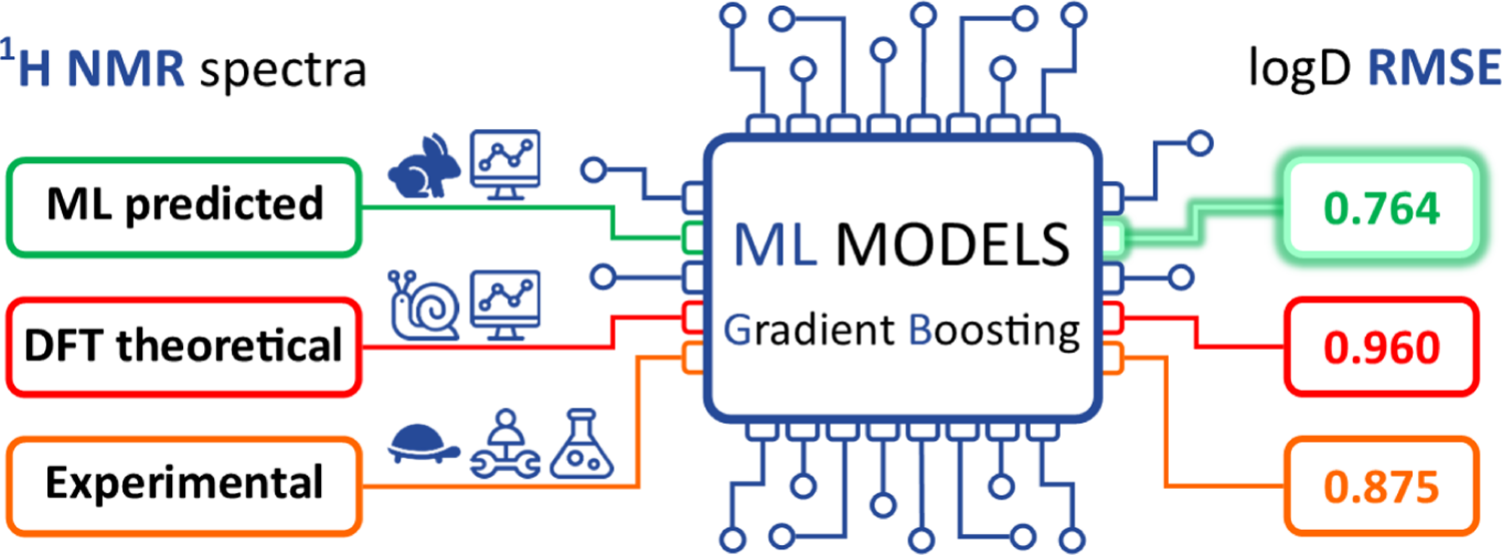

This study presents a novel approach to ^1^H NMR-based machine learning (ML) models for predicting logD using computer-generated ^1^H NMR spectra. Building on our previous work, which integrated experimental ^1^H NMR data, this study addresses key limitations associated with experimental measurements, such as sample stability, solvent variability, and extensive processing, by replacing them with fully computational workflows. Benchmarking across various density functional theory (DFT) functionals and basis sets highlighted their limitations, with DFT-based models showing relatively high RMSE values (average CHI logD of 1.12, lowest at 0.96) and extensive computational demands, limiting their usefulness for large-scale predictions. In contrast, models trained on predicted ^1^H NMR spectra by NMRshiftDB2 and JEOL JASON achieved RMSE values as low as 0.76, compared to 0.88 for experimental spectra. Further analysis revealed that mixing experimental and predicted spectra did not enhance accuracy, underscoring the advantage of homogeneous datasets. Validation with external datasets confirmed the robustness of our models, showing comparable performance to commercial software like Instant JChem, thus underscoring the reliability of the proposed computational workflow. Additionally, using normalized RMSE (NRMSE) proved essential for consistent model evaluation across datasets with varying data scales. By eliminating the need for experimental input, this workflow offers a widely accessible, computationally efficient pipeline, setting a new standard for ML-driven chemical property predictions without experimental data constraints.

## Introduction

In the current state of computational chemistry, predictive models based on two-dimensional (2D) molecular descriptors have reached a plateau of performance. Traditional 2D descriptors, such as molecular fingerprints, encode structural information about the presence or absence of specific substructures and functional groups.^1−4^ While effective, these descriptors are inherently limited in capturing the complexity of the chemical information. Specifically, they fail to provide insight into the molecular environment, conformational dynamics, nuclei near the nanoparticle, and intermolecular interactions. As a result, advances in predictive modeling using 2D descriptors have become incremental, with most recent developments focusing on refining existing methods rather than introducing fundamentally new approaches.^5−7^ Many predictive models developed using these descriptors rely on traditional machine learning algorithms such as Logistic Regression (LR), Random Forest (RF), and Support Vector Machines (SVM), with RF often considered the “gold standard”.^8,9^ However, the performance of these models is highly dependent on the descriptors chosen for training, and it has been shown that models based solely on molecular fingerprints often perform suboptimally.^10,11^ Furthermore, limited attention has been given to newer, state-of-the-art machine learning algorithms like XGBoost and LightGBM, which have demonstrated great potential for predicting molecular properties.^12,13^ Despite the development of graph-based methods, which are reported to outperform descriptor-based approaches, this remains a topic of ongoing debate, highlighting the need for further exploration of new methodologies to move beyond the limitations of 2D descriptors.^6^

In contrast, Nuclear Magnetic Resonance (NMR) spectra offer a notably richer source of information that can be leveraged as a molecular fingerprint. NMR spectra not only reflect the chemical shifts associated with specific nuclear environments but also provide indirect data about molecular conformation, electron distribution, and intermolecular interactions such as hydrogen bonding and steric effects. This additional layer of data makes NMR spectra a valuable and irreplaceable tool for capturing the subtle nuances of molecular behavior that are often missed by traditional molecular fingerprints. Using NMR spectra as a basis for predictive modeling makes it possible to account for both intra- and intermolecular interactions, offering a more comprehensive representation of a molecule’s properties.^14^

Our previous work demonstrated the potential of integrating ^1^H NMR data with machine learning (ML) to predict the distribution coefficient logD.^15^ The logD is an essential parameter for quantifying lipophilicity, as it accounts for ionization, making it more relevant for drug research since most drugs contain ionizable groups. Lipophilicity influences key physicochemical properties of drugs, including absorption, distribution, metabolism, and toxicity. Excessive lipophilicity may increase toxicity risks, while low lipophilicity can limit absorption and metabolism. Accurate determination of logD is crucial for assessing the pharmacokinetic properties and safety of potential drug candidates.

In that study, we benchmarked several machine learning algorithms, including Support Vector Regression (SVR), Gradient Boosting, and AdaBoost, against traditional 2D molecular fingerprints such as MACCS, Klekota-Roth, Extended-Connectivity Fingerprints (ECFPs), RDKit Fingerprints, and Molecular Descriptors. The ^1^H NMR-based models had similar outputs to the fingerprint-based models, with the Gradient Boosting model combined with 10-fold cross-validation (10CV) achieving the highest accuracy (0.87). However, the major limitation of the proposed approach was its reliance on experimental ^1^H NMR data, which are both time-consuming and resource-intensive to collect. In addition, the inherent variability in experimental conditions—such as solvent effects, sample purity, and spectrometer settings—introduced noise into the datasets, negatively impacting the model’s generalizability. The study also highlighted that experimental data availability was a bottleneck, as collecting high-quality NMR spectra for large datasets is labor-intensive and subject to various experimental inconsistencies. These challenges underscored the need for alternative approaches to utilize theoretical and predicted spectra or mixed datasets that combine experimental and generated spectral data to overcome these limitations.

In this study, we build upon our earlier approach by utilizing theoretical and predicted ^1^H NMR spectra to overcome the reliance on experimental data. We examine the feasibility of supplementing or replacing experimental datasets with generated spectra in the training of logD predictive models. By systematically comparing experimental and generated spectral data obtained from different sources, we aim to determine whether theoretically generated or predicted spectra can offer comparable accuracy and reliability. To achieve this, we benchmark a broad range of Density Functional Theory (DFT) functionals and basis sets, as well as ML-based methods, to ensure a comprehensive evaluation of spectrum generation. Additionally, we assess the performance of models trained on mixed datasets—combining experimental and generated spectra in specific ratios. The objective of this work is to develop a more versatile and accessible methodology that expands the potential applications of NMR-based predictions while minimizing reliance on resource-intensive experimental procedures. By leveraging theoretical and predicted spectral data, we aim to provide robust predictive models, enabling a more efficient exploration of the chemical space.

## Materials and Methods

### Compound Dataset

A total of 754 chemical compounds, selected from the Celon Pharma internal database, were used in this study. The selection criteria included a broad diversity of chemical structures, encompassing various functional groups and molecular cores, as well as the sufficient quality of the ^1^H NMR spectra and the availability of experimental logD values. The values were measured chromatographically at three pH points: 2.6, 7.4, and 10.5. Structural diversity was assessed through automatic hierarchical clustering of the dataset, using the Tanimoto metric with ECFP4 fingerprints for similarity analysis and a complete linkage method to group the compounds. A more detailed description of the dataset can be found in our previous work.^15^

### Experimental Determination of LogD

CHI logD and Chrom logD are chromatographically derived parameters used to quantify the lipophilicity of chemical compounds, serving as practical alternatives to conventional logD. While traditional logD measures the distribution coefficient of a compound between octanol and water phases at a specific pH, CHI logD and Chrom logD are determined by using high-performance liquid chromatography (HPLC) techniques. The complete characterization of these two parameters, their interdependence, and the methodology for their measurement and determination have been described in previous studies.^15,16^

### Experimental ^1^H NMR Spectra

^1^H NMR spectra were acquired on a JEOL JNM-ECZS 400 MHz, JEOL JNM-ECZR 600 MHz, Bruker DRX 500 MHz, and Varian Inova 300 MHz spectrometers. Spectra were measured in DMSO-*d*_6_ or CDCl_3_ solution at 298 K temperature. Signals were referenced to DMSO-*d*_6_ with a chemical shift defined at 2.50 ppm or CDCl_3_ with a chemical shift of 7.26 ppm. Alongside the standard analysis and interpretation of the ^1^H NMR, chemical shifts were listed for each hydrogen atom to perform a related DFT benchmark so that the final number of chemical shifts equaled the number of hydrogen atoms.

The detailed procedure for processing and preparing experimental ^1^H NMR spectra as inputs for training machine learning models is described in a previous study.^15^ We selected the most optimal and universally applicable approach based on the findings. The data were reduced using the Bucket Integration method, down to 500 points from the original 16,384, and then normalized within the range of 0 to 1000.

### Theoretical and Predicted ^1^H NMR Spectra Generation

In this study, we utilized three fundamentally different sources of computer-generated ^1^H NMR spectra, each varying in spectral resolution, computational cost, and processing time. For training machine learning models, theoretical spectra were generated using a quantum-mechanics approach, specifically Density Functional Theory, across various levels of basis sets and functionals. In addition, JEOL JASON^17^ software was employed to predict spectra that closely mimic real ^1^H NMR spectra. The third method involved a standalone chemical shift predictor for ^1^H NMR spectra, developed using HOSE codes and based on the NMRshiftDB2 database.^18−20^

All quantum-chemical calculations were performed using the Gaussian 16 (G16) software package.^21^ The molecular geometries were optimized using DFT,^22−25^ with five different functionals: B3LYP-D3BJ,^26−28^ CAM-B3LYP-D3BJ,^29^ M06-2X-D3,^30,31^ PBEPBE-D3BJ,^32,33^ and wB97XD.^34^ The D3 version of Grimme’s dispersion correction with Becke-Johnson (BJ) damping was applied to account for long-range electron correlation effects, improving the accuracy of density functional theory calculations by correcting for dispersion interactions. These were tested with six basis sets: 6-31G(2d), 6-311+G(2d,p) – Pople basis sets,^35,36^ cc-pVDZ, cc-pVTZ – Dunning’s correlation-consistent basis sets,^37,38^ def2-SVP, and def2-TZVP – Karlsruhe basis sets.^39,40^ Solvent effects were included using the polarizable continuum model (PCM)^41^ for chloroform (CDCl_3_) and dimethyl sulfoxide (DMSO) to replicate experimental conditions.

To ensure consistency in geometry optimization, the following convergence thresholds were applied across all functionals and basis sets:

- Maximum Force: 4.5 × 10^–4^ Ha/Bohr,
- RMS Force: 3.0 × 10^–4^ Ha/Bohr,
- Maximum Displacement: 1.8 × 10^–3^ Bohr,
- RMS Displacement: 1.2 × 10^–3^ Bohr.

These parameters ensured accurate and well-converged molecular structures suitable for subsequent NMR calculations.

^1^H NMR shielding tensors were computed using the gauge-independent atomic orbital (GIAO) method within the same functional and basis set framework.^42−44^ The calculated ^1^H NMR chemical shifts were referenced to tetramethylsilane (TMS) as an internal standard, using calculated values from the same computational set for comparison. Shielding tensors are intrinsic properties of the molecule and are independent of the reference standard and the resonance frequency of the spectrometer. Conversion to chemical shifts was necessary to compare the calculated shielding values to the experimental data. No explicit signal assignment was used for comparison between calculated and experimental spectra, as a semiautomated analysis was required. The analysis was conducted by comparing the occurrence of successive signals in both experimental and theoretical ^1^H NMR spectra. The chemical shifts of signals originating from the same nuclei in a structural sense were not compared; only the order of their appearance in the spectra was evaluated. The benchmark simulated the approach of machine learning algorithms in reading input data without a specific assignment of signals to structures. The root-mean-square error (RMSE) was calculated without individual signal matching to evaluate the overall agreement between the calculated and experimental shifts for each hydrogen. The heatmaps and boxplots were generated by our Python script,^45^ utilizing the Matplotlib^46^ and Seaborn^47^ libraries, to visualize the performance of different functional and basis set combinations.

Following the benchmark analysis, the CAM-B3LYP/6–311+G(2d,p) combination was used to calculate the ^1^H NMR spectra for the entire molecular library. The PCM model was applied for CDCl_3_ as a solvent. For structures containing heavy atoms, the GenECP with pseudopotential for heavy atoms (def2-TZVP) was used to account for relativistic effects.

All calculations were performed on the Ares supercomputer at the HPC Center: ACK Cyfronet AGH, utilizing 24 cores per job, ensuring efficient parallel processing and reducing computation time. Computation times for each job were extracted directly from the Gaussian log files, allowing for an assessment of the computational efficiency alongside the accuracy.

The second computer-generated ^1^H NMR spectra method used was a tool implemented in JEOL JASON software 4.0.^17^ Spectra were predicted from structures saved in .mol files. The predictions were performed at a frequency of 600 MHz, with a line broadening factor of 1.0 Hz and 64K points across a spectral window from 0 to 12 ppm. All generated spectra were then subjected to postprocessing using the resample function, with the range set from −1 to 12 ppm and 16,384 points. Each spectrum generation took several seconds and required manual processing and saving of the results. The spectrum produced by the JASON software is a fully representative ^1^H NMR spectrum, in which the signals not only appear at the defined frequencies but also include relative integration and multiplet structure, allowing for the reading of coupling constants. The spectrum lacks only the signals of the solvent, internal standard, and typical impurities, such as water.

The last method used for generating predicted ^1^H NMR spectra involved the standalone predictor1h.jar,^48^ which contains a Java class org.openscience.nmrshiftdb, a prediction tool, and a CSV file. This file includes all HOSE codes and corresponding shift values from NMRshiftDB2.^18−20^ The predictor was employed in conjunction with the Chemistry Development Kit (CDK) version 2.9^49^ and a modified script published on the project’s SourceForge page^48^ which is provided in the Supporting Information of this publication. Properly prepared .mol files of the compound structures were used as input for the predictions. Loading structures as flat coordinates or 3D structures occasionally caused stereochemistry assessment errors on certain atoms. To address this, structures were prepared by loading SMILES codes to a Python script, then 3D coordinates were generated using the Python RDKit library,^50^ which were then flattened and saved to files already in the form of 2D coordinates. The flattening process involved zeroing out the z-coordinates. These prepared structures worked seamlessly with the ^1^H NMR predictor.

### Generating Spectra from Predictions

The results from both DFT calculations and the predictions using the NMRshiftDB2-based tool are not conventional ^1^H NMR spectra in the form of matrices with tens of thousands of frequency-intensity pairs. Instead, they provide only a list of chemical shifts corresponding to individual simulated nuclei. This poses a challenge for generating inputs for machine learning models, as the feature space for each vector must have the same number of components, which is impossible for compounds with varying numbers of nuclei. To generate standardized ^1^H NMR spectra, a modified bucket integration methodology^15^ was employed, which has been successfully used for dimensionality reduction tasks. A Python script was developed that divides the chemical shift range from −1 to 14 ppm into 500 equal bins (buckets), with each bucket initially assigned a value of 0. The script then analyzed the provided array of frequencies (chemical shifts) from the predicted spectra. If a chemical shift value from the list fell within a specific bucket, that bucket’s value increased by 1. For example, three identical protons from a methyl group would generate a bucket with a corresponding chemical shift and an intensity of 3. In contrast, a methylene group would produce one bucket with an intensity of 2. These pseudo-NMR spectra, now formatted as matrices with equal numbers of chemical shift-intensity pairs, were ready to be used for training machine learning models.

### Machine Learning

To prepare inputs from computer-generated ^1^H NMR spectra for training machine learning models, we used the Python scripts developed in our previous study.^15^ Spectra produced by JASON were normalized in the same range as experimental spectra, i.e., from 0 to 1000. DFT spectra were normalized due to their inherent structure in a range from 0 to 1. Similarly, spectra generated by the NMRshiftDB2 predictor did not require normalization, as all values were integers and multiples of 1. As for model selection, Gradient Boosting was chosen for its ability to handle data subjected to different dimensionality reduction and normalization methods, as confirmed by previous research.^15^ For model training and evaluation, a 10CV (10-fold cross-validation) was applied.

### Assessment of Predictive Model’s Performance

RMSE, namely the root-mean-square error (eq 1), is a common evaluation metric in machine learning used to measure the difference between predicted  and actual values *y*_*i*_ in regression tasks. It is calculated as the square root of the average of the squared differences between predictions and true values, providing a measure of the model’s prediction accuracy. Low RMSE values indicate better model performance as they reflect smaller errors and a closer fit to the actual values of predicted parameters.
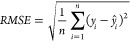
1

### Mixing Spectra

The random mixing of predicted/theoretical with experimental ^1^H NMR spectra was performed ten times for each pair of spectral datasets in proportions of 20%, 40%, 60%, and 80%. This mixing scheme was repeated three times: once for the theoretical spectra obtained from DFT calculations, once for those from the NMRshiftDB2 predictor, and once for the spectra predicted by using the JASON software. The resulting mixed datasets were then used as inputs for training machine learning models with 10CV as the evaluation method.

## Results and Discussion

### Comparison of DFT Levels for Accurate ^1^H NMR Predictions

A comprehensive benchmark was performed to assess the accuracy of quantum mechanical methods for calculating ^1^H NMR spectra by using a broad range of DFT functionals and basis sets. The calculated ^1^H NMR chemical shifts were referenced to tetramethylsilane (TMS), and RMSE was used to assess the overall agreement between the calculated and experimental shifts. A semiautomated approach was applied, where no explicit signal assignment was made, allowing for an efficient comparison between calculated and experimental spectra. This method provided an unbiased evaluation of how well each functional and basis set combination captured the general accuracy of the chemical shifts. The selected functionals represent a diverse set of approaches, from hybrid functionals like B3LYP and CAM-B3LYP, which incorporate both local exchange-correlation and a portion of exact Hartree–Fock exchange, to dispersion-corrected functionals such as wB97XD and PBEPBE-D3BJ, designed to account for long-range interactions. M06-2X, a meta-hybrid functional specifically designed for thermochemistry, kinetics, and noncovalent interactions, was also included due to its suitability for molecules with varying electronic properties. The basis sets were selected to provide a range of computational efficiency and accuracy, ensuring compatibility with the chosen functionals for both weakly and highly polar solvent environments. The Pople-type basis sets, due to their efficiency, are widely used in computational chemistry,^51^ particularly for small and medium-sized molecules. Meanwhile, the correlation-consistent basis sets from Dunning are designed to systematically converge electron correlation effects, offering higher accuracy at greater computational expense. Finally, the Karlsruhe def2-SVP and def2-TZVP basis sets were selected for their optimization with DFT methods and their ability to model transition metals and heavy atoms, making them ideal for a broad class of organic and organometallic compounds.

The spectra were divided based on the solvent used to dissolve the compounds. CDCl_3_, being a weakly polar solvent, primarily engages in dipole–dipole interactions,^52^ which can be reasonably approximated by PCM through its dielectric constant. In contrast, DMSO is a strongly polar solvent, introducing not only dipole–dipole interactions but also more complex solute–solvent interactions, such as hydrogen bonding.^53^ PCM does not directly model specific solute–solvent hydrogen bonding or other nonelectrostatic interactions. Thus, in highly polar solvents like DMSO, where these interactions are significant, PCM might be an incomplete model, leading to less accurate calculations of NMR chemical shifts and consequently higher RMSE in predicting experimental ^1^H NMR spectra.^54−56^

**Figure 1:**
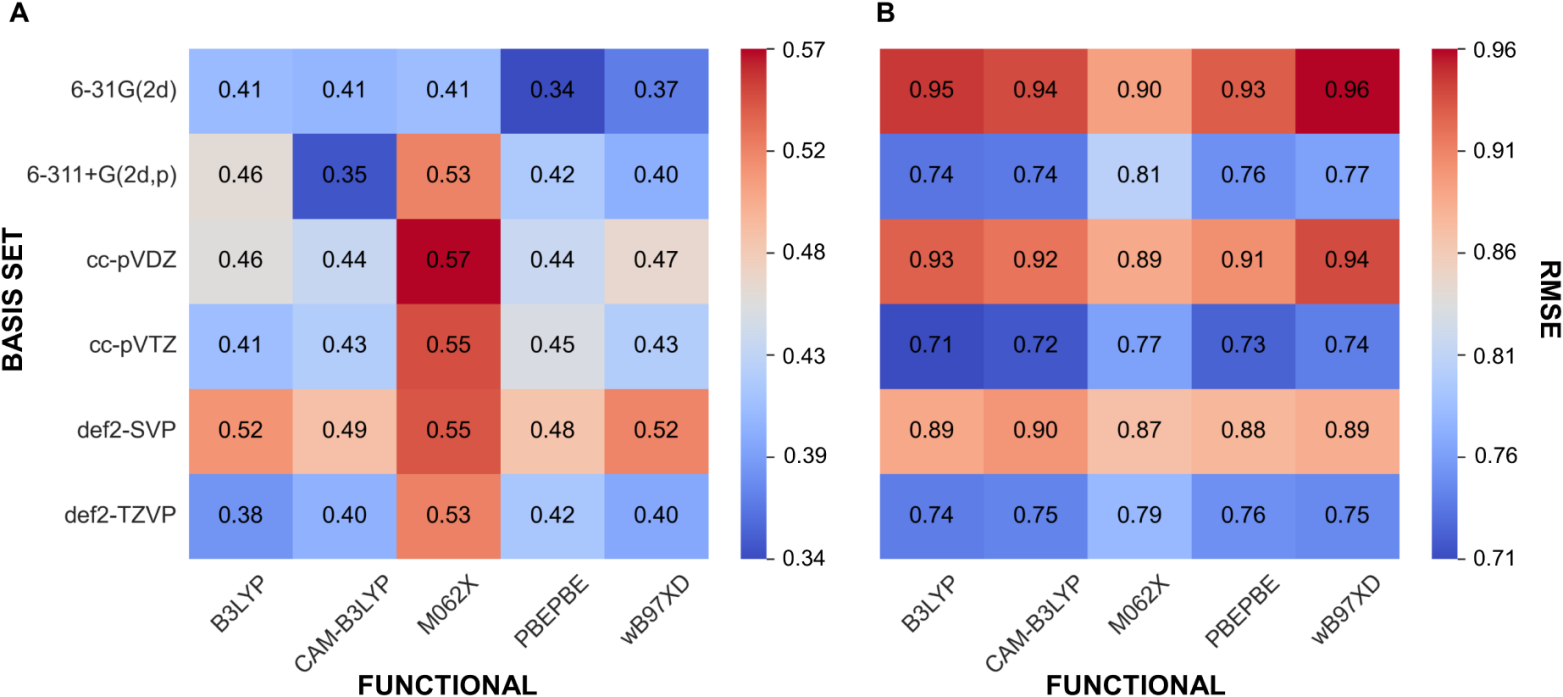
Heatmaps illustrating the mean RMSE for theoretical ^1^H NMR spectra generated using various functionals and basis sets compared to experimental spectra measured in CDCl_3_ (A) and DMSO (B) solvents. Lower RMSE (blue) indicates better agreement with experimental data, while higher RMSE (red) represents greater deviations.

The performance of different DFT methods combined with various basis sets in reproducing ^1^H NMR spectra for CDCl_3_ and DMSO showed distinct trends due to the nature of the solvent environments (Figure 1). In the case of CDCl_3_, a weakly polar solvent, the overall RMSE values remained relatively low across most functionals and basis sets. Notably, the lowest RMSE values were obtained with simpler basis sets, such as 6-31G(2d), as seen with PBEPBE (0.345) and CAM-B3LYP (0.350). These results suggest that the solute–solvent interactions in CDCl_3_, primarily dipole–dipole, are sufficiently modeled by simpler basis sets, and the additional polarization and dispersion corrections do not substantially improve spectra reproducing accuracy. This finding highlights the efficiency of smaller basis sets in capturing the essential interactions in such a solvent where long-range electron correlation effects play a minor role. Interestingly, more complex basis sets like def2-TZVP did not outperform the smaller sets in CDCl_3_, with functionals such as wB97XD (0.396) and B3LYP (0.384) yielding only slightly better results. Functionals without explicit dispersion corrections, such as M06-2X, exhibited slightly higher RMSE values overall, particularly with larger basis sets such as cc-pVTZ (0.548). This highlights that while CDCl_3_ does not require extensive corrections for electron correlation and long-range interactions, functionals with minimal dispersion corrections are still effective. This trend suggests that while functionals with dispersion corrections might refine the geometry, their impact on chemical shift predictions in CDCl_3_ remains minimal due to the solvent’s weak polarization environment. Moreover, additional polarization in basis sets, such as def2-SVP and cc-pVDZ, yielded higher RMSE values, especially for M06-2X (0.545 and 0.569, respectively). This reinforces the idea that introducing extra polarization in the basis sets does not provide a proportional benefit for CDCl_3_. Instead, the inherent simplicity of the solvent’s interactions appears to be well-represented by smaller, less computationally demanding configurations. Consequently, this solvent environment provides a compelling case for the practical use of minimal basis sets and standard functionals without extensive corrections, particularly for computational efficiency in large-scale studies.

In contrast, DMSO, a highly polar solvent, presented a more challenging environment for accurate ^1^H NMR calculations, with consistently higher RMSE values across all functionals and basis sets. The lowest RMSE values in DMSO were still higher than those in CDCl_3_, with B3LYP/cc-pVTZ (0.715) and CAM-B3LYP/cc-pVTZ (0.721) leading the performance, followed closely by PBEPBE/cc-pVTZ (0.728). These results indicate that in DMSO, the strong solute–solvent interactions require more complex functionals and basis sets to achieve comparable accuracy. The trend was particularly evident when comparing the performance of the 6-31G(2d) basis set between CDCl_3_ and DMSO. While it performed well in CDCl_3_ (e.g., PBEPBE/6-31G(2d) at 0.345), it yielded significantly higher RMSE values in DMSO (e.g., PBEPBE/6-31G(2d) at 0.933). This reflects the more complex polar and hydrogen bonding interactions in DMSO, which are not adequately captured by simpler basis sets. Adding extra polarization and dispersion functions increases the prediction accuracy in DMSO. In contrast, the M06-2X functional showed consistently higher RMSE values in DMSO, with the best result being 0.767 with cc-pVTZ. This suggests that M06-2X, while effective for thermochemistry and kinetics, may not capture the necessary long-range interactions in highly polar solvents such as DMSO. Additionally, for basis sets such as def2-SVP, the RMSE values were significantly higher in DMSO, with B3LYP (0.891) and CAM-B3LYP (0.903) performing similarly. These higher RMSE values may suggest that the polarization functions in def2-SVP do not fully capture complex solute–solvent interactions in DMSO, leading to less accurate predictions. The detailed distribution of RMSE values for each functional and corresponding basis sets is provided in the (Figures S1 and S2).

To identify the best DFT approach for further analysis, each set was ranked based on mean RMSE values in both CDCl_3_ and DMSO solvents. The rankings were determined separately for each solvent and then summed to provide an overall performance score. Based on this combined ranking, the two top levels of theory—CAM-B3LYP + 6–311 + G(2d,p) and B3LYP + def2-TZVP—were selected as they consistently showed the best reproduction performance of ^1^H NMR spectra for both solvents (Table S1).

Statistical analysis was used to compare the performance of the def2-TZVP and 6–311+G(2d,p) basis sets across both solvents (CDCl_3_ and DMSO) using a paired *t*-test. These two basis sets were selected for statistical comparison, as they demonstrated the lowest average RMSE values in the benchmarking analysis, making them the most relevant candidates for further evaluation. For CDCl_3_, the results showed no statistically significant difference in the RMSE between the two basis sets (*p* = 0.775), indicating that both basis sets perform similarly. Analogously, for DMSO, the *t*-test results also indicated no significant difference between the two basis sets (*p* = 0.348). Given the lack of significant statistical difference in RMSE between basis sets in both solvents and the fact that 6-311 + G(2d,p) needs less computational time (Table S2), this basis set was chosen for the calculations of the entire molecular library in combination with the CAM-B3LYP functional. When the combination of 6-311+G(2d,p) (for H, N, C, and F atoms) and def2-TZVP (for I atom) basis sets was used for compounds containing iodine, the theoretical ^1^H NMR spectra showed unexpected shifts for protons located near the iodine atom in the molecular structure. These protons were affected because iodine’s large core electron count leads to significant relativistic effects that alter the electron density around the atom. Standard basis sets without pseudopotentials struggle to account for these effects, resulting in incorrect shielding constants for neighboring protons. By applying the effective core potentials option in Gaussian (GenECP), the relativistic effects of iodine’s core electrons are properly modeled, restoring accurate ^1^H NMR calculations for the protons in its vicinity.^57,58^

### Characteristics of the Generated ^1^H NMR Spectra

**Figure 2:**
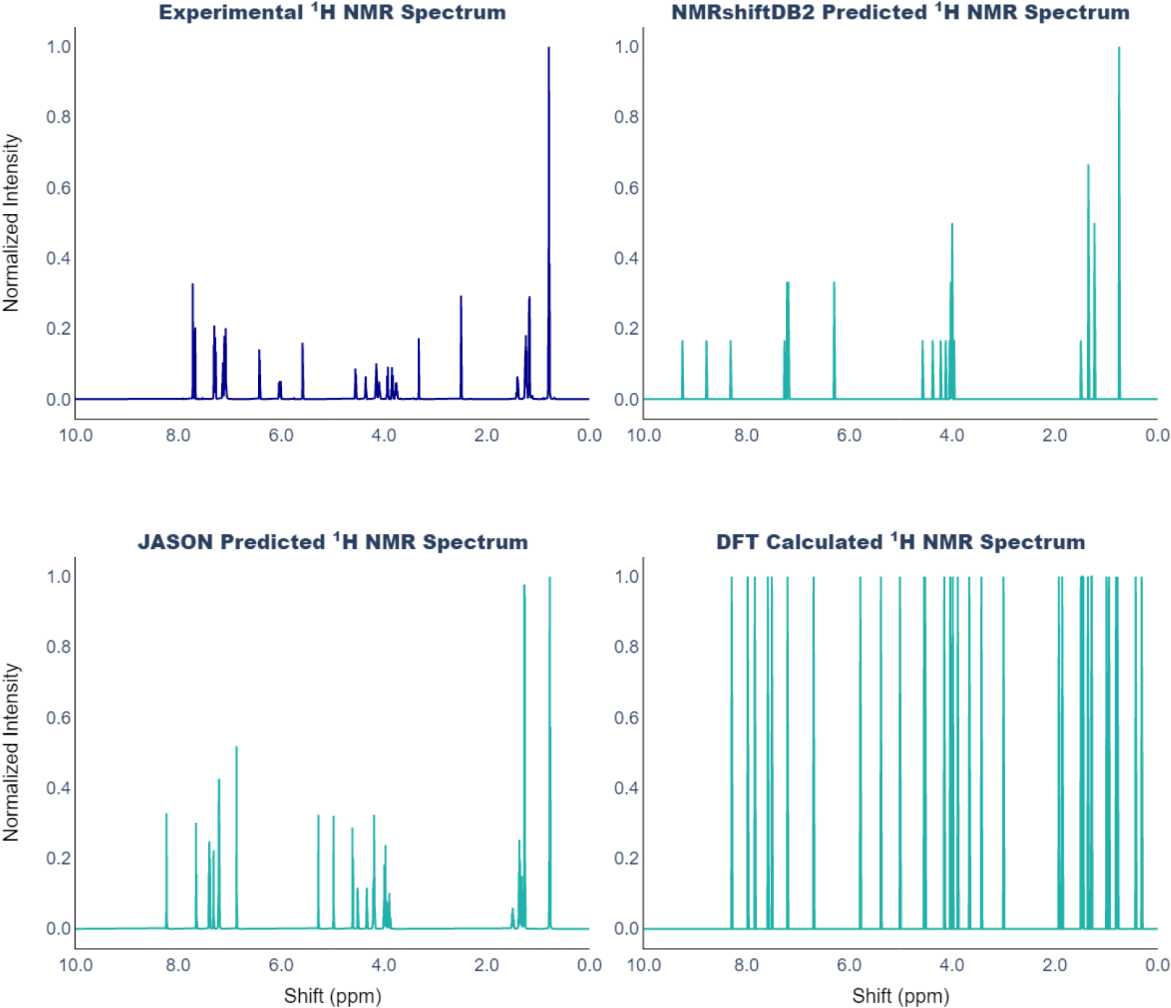
Comparison of three methods for generating ^1^H NMR spectra alongside the experimental. All spectra shown were generated for the same compound. The intensity for each spectrum has been normalized.

The obtained computer-generated ^1^H NMR spectra exhibited varying characteristics and structures, as shown in Figure 2. Among the methods, the spectra produced by the JEOL JASON software were the most similar to the experimental ^1^H NMR spectra. This prediction accurately captured multiplets, chemical shifts, and natural relative integration, which allowed for inverse Fourier transformation to calculate an FID (Free Induction Decay) in the time domain.

In contrast, spectra obtained through DFT calculations and the NMRshiftDB2 predictor lacked traditional spectral characteristics. Instead, these methods produced only a list of chemical shifts, which were transformed into a spectrum-like representation using bucketing. In the case of the NMRshiftDB2 predictor, chemically equivalent protons were assigned identical chemical shifts, meaning that the pseudo-^1^H NMR spectrum preserved the relative integration of the signal groups. However, in the DFT-calculated spectra, relative integration was absent, with each signal having an integral value of 1. This discrepancy arises because DFT calculations are performed in a stationary state, without dynamic averaging of equivalent nuclei. For example, in a DFT-based spectrum, a methyl group contains three distinct signals, each with different shifts and an integral value of 1, rather than a single signal with an integration value of 3, as seen in experimental or ML-predicted spectra.

NMR prediction methods have an advantage over experimental data as they are not susceptible to noise resulting from sample impurities, solvent traces, or incorrect identification of the sample’s contents. Additionally, preprocessing of the experimental spectra was conducted automatically without segregating the spectra based on specific solvent groups. Instead, all regions where solvent signals were likely to appear were removed from the spectra. This approach was justified by the large size of the input dataset and the need for automated preprocessing procedures. For large experimental datasets, complete and reliable characterization of each spectrum becomes practically unfeasible. In the case of experimental ^1^H NMR spectra, results are also influenced by factors related to different spectrometers, measurement parameters, and variability arising from the work of different operators. Unlike experimental, theoretical, or predicted ^1^H NMR spectra form a homogeneous dataset that contains only information derived from the defined chemical structures, devoid of disturbances and unnecessary noise. Additionally, the DFT method is characterized by a considerably higher computational cost, with the time required to process a single compound measured in hours, compared to just a few seconds needed for the other two methods. The biggest advantage of the NMRshiftDB2-based method is its complete automation of spectra generation based on SMILES codes of the compound, whereas spectra in the JASON program were generated manually using structures saved in .mol files. It is worth noting that the entire process in JASON can now be automated using the Python library BeautifulJASON.^59^

### Comparison of Experimental and Generated ^1^H NMR Spectra

To compare the potential and usefulness of different types of ^1^H NMR spectra, a series of logD predictive models were trained based on the ^1^H NMR spectra source and Gradient Boosting algorithm.^60^ Gradient Boosting consistently showed strong robustness against variations in data preprocessing, producing reliable results focusing on the intrinsic properties of the data.^15^ For model training and evaluation, 10CV (10-fold cross-validation) was employed as a method with lower computational cost compared to that of Leave-One-Out (LOO) cross-validation. Both data-splitting techniques yielded similar performance for the machine learning algorithms. Using LOO, the RMSE values were obtained in the previous study as 0.66 for the SVR model and 0.67 for the Gradient Boosting model. In contrast, 10CV resulted in RMSE values of 0.88 for SVR and 0.87 for Gradient Boosting. Gradient Boosting was ultimately chosen due to its straightforward integration with GPU-based CUDA computations, which enhances computational efficiency.

**Figure 3:**
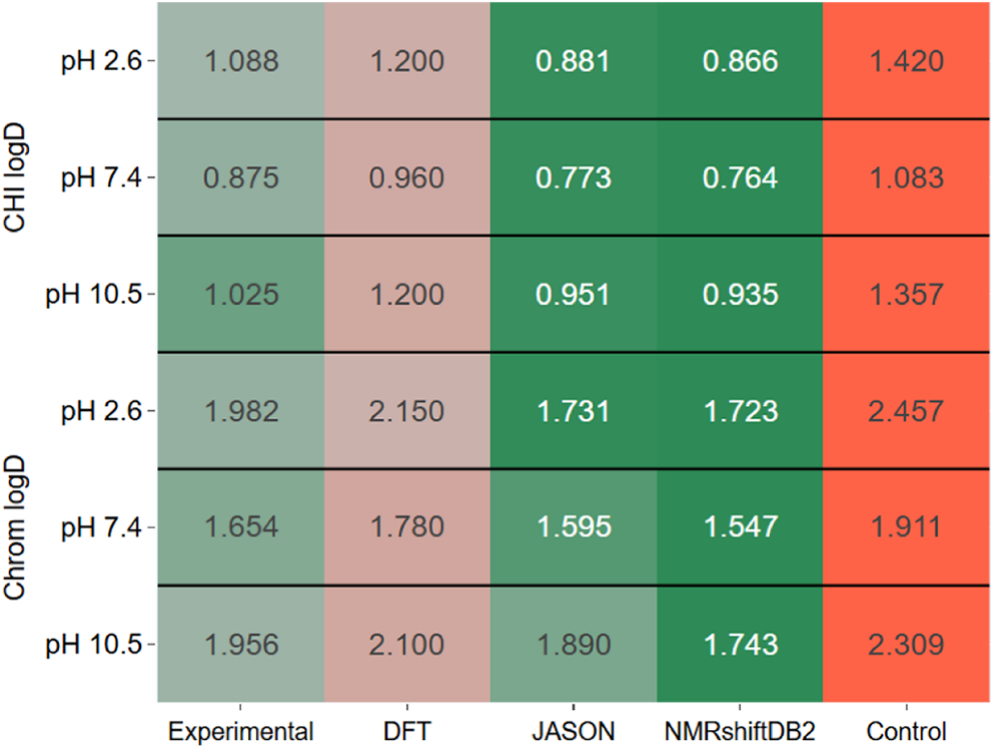
Comparison of RMSE values obtained by 10CV for the machine learning models for both experimental and theoretical/predicted spectra. A negative control column was added, where models were trained on hashed label vectors instead of spectra. The values are organized in rows according to specific logD parameters. The best-performing models are highlighted in green, whereas the worst-performing models are marked in red.

The analysis of the results reveals three main patterns in RMSE values following model training, as illustrated in Figure 3. The highest RMSE values for each of the modeled parameters across different pH levels were observed for DFT-based spectra, which is indicated by the red column (i.e., the worst-performing models in a given series) in Figure 3. On average, RMSE values for DFT-based spectra were 17% higher for the Chrom logD and 23% higher for the CHI logD compared to the best-performing method available for each parameter. Moreover, these values were 7% and 11% higher, respectively, compared to the next best-performing approach within the given series.

The second pattern corresponds to experimental spectra, which provide stable and moderate RMSE values, positioning them between the DFT-based and the ML-based spectra (JASON and NMRshifDB2).

The third pattern comprises results obtained for the spectra produced by the JASON software and predictions based on the NMRshiftDB2 database. For the CHI logD, the model performances for both methods were nearly identical. A minor deviation was observed for the Chrom logD, where NMRshiftDB2-based models outperformed JASON-based models by 8.5%, 3.1%, and 0.6% at pH values of 10.5, 7.4, and 2.6, respectively. Despite these minor differences, the lowest RMSE values were consistently achieved using NMRshiftDB2-based spectra.

A comparison of RMSE metric values for models trained on different sources of computer-generated and experimental spectra (Figure 3) highlights distinct trends related to the complexity of the input data. Notably, DFT-based spectra stand out due to the absence of relative signal integration, and the representation of molecules in a stationary state significantly diminishes the prediction accuracy of logD. In contrast to DFT, spectra generated using the NMRshiftDB2-based and JASON-based methods represent kinetically averaged molecular structures, allowing for the relative integration of signal groups in the spectra. In addition, the lower values of RMSE for ML-based methods may be due to the lack of noise relative to the experimental spectra. Noise is defined as additional signals from impurities and erroneous records in the structure-spectrum NMR database.

To further validate the robustness of the models and rule out potential shortcut learning or data leakage, a control experiment was conducted. In this experiment, models were trained on datasets completely devoid of meaningful spectral information, using hashed label vectors instead of spectra. The results (Figure 3) revealed that predictive performance on these meaningless datasets was approximately 40% worse than on models trained with NMR-based spectral data. This substantial decrease in accuracy strongly supports the conclusion that the models extract relevant chemical relationships from the spectra rather than relying on spurious correlations or unintended artifacts. These findings further underscore the critical role of spectral representation in achieving accurate logD predictions.

**Figure 4:**
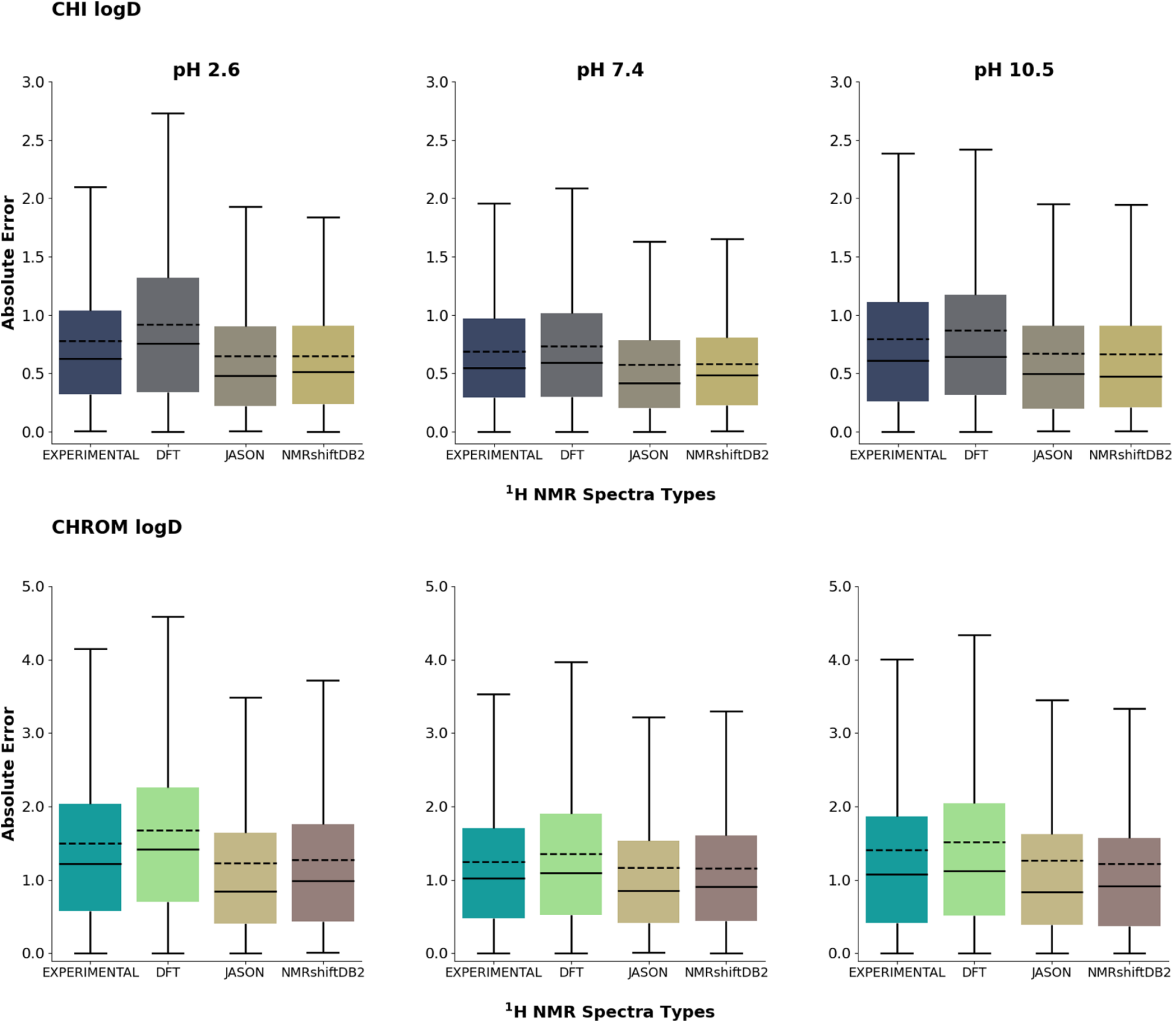
Boxplots of absolute errors between computer-generated and experimental predictions of CHI logD (top) and Chrom logD (bottom) at different pH. In each plot, the first box corresponds to experimental ^1^H NMR spectra, whereas the remaining ones are the methods used to obtain the generated ^1^H NMR spectra. In each box, the horizontal dashed line describes the mean value, while the solid line refers to the median. Charts with marked outliers can be found in the Supporting Information.

The analysis of the mean logD prediction error and its distribution for the CHI logD and Chrom logD models (Figure 4) indicates that the models performed the worst when applied to DFT-based and experimental spectra. The medians of the absolute errors were relatively high—0.76, 0.59, and 0.64 for CHI logD at pH 2.6, 7.4, and 10.5, respectively—and the distributions of the errors were broad, with mean values of 0.92, 0.73, and 0.87 for the same conditions. The medians of the absolute errors for JASON-based predictions were 0.48, 0.42, and 0.50, with mean absolute errors of 0.65, 0.58, and 0.67 for CHI logD at pH 2.6, 7.4, and 10.5, respectively. Similarly, the NMRshiftDB2-based models produced medians of 0.51, 0.48, and 0.47, with corresponding mean absolute errors of 0.64, 0.58, and 0.66. For models derived from experimental ^1^H NMR data, the medians of the absolute errors were 0.62, 0.55, and 0.61, while the mean values of the absolute errors were slightly higher, at 0.78, 0.69, and 0.80 for CHI logD at pH 2.6, 7.4, and 10.5, respectively. The improved performance of models using input data from the NMRshiftDB2 predictor may be attributed to the structure of the feature vectors, which are composed primarily of zeros and discrete values, typically integer multiples of 1. On the other hand, JASON-based spectra include feature vectors with floating-point numerical values, similar to those from experimental spectra methods, which could contribute to the observed differences in predictive performance. Although the simplification of input data might enhance model efficiency, it is not the sole determinant of model effectiveness, as evidenced by the poor performance of models using DFT-based spectra. Despite DFT-calculated ^1^H NMR spectra having binary feature vectors, similar to NMRshiftDB2 predicted spectra, the models based on DFT data exhibited poor predictive capabilities.

Founded on these results, it can be concluded that critical factors for the accurate prediction of logD based on theoretical ^1^H NMR spectra are the resonance frequencies of individual nuclei, the relative integration of homogeneous signal groups, and the absence of interference from contaminants and external effects. Conversely, the multiplet structure, resulting from coupling constants, along with the shape and width of the signals, has minimal impact on the model’s predictive performance.

### Applicability Domain

**Figure 5:**
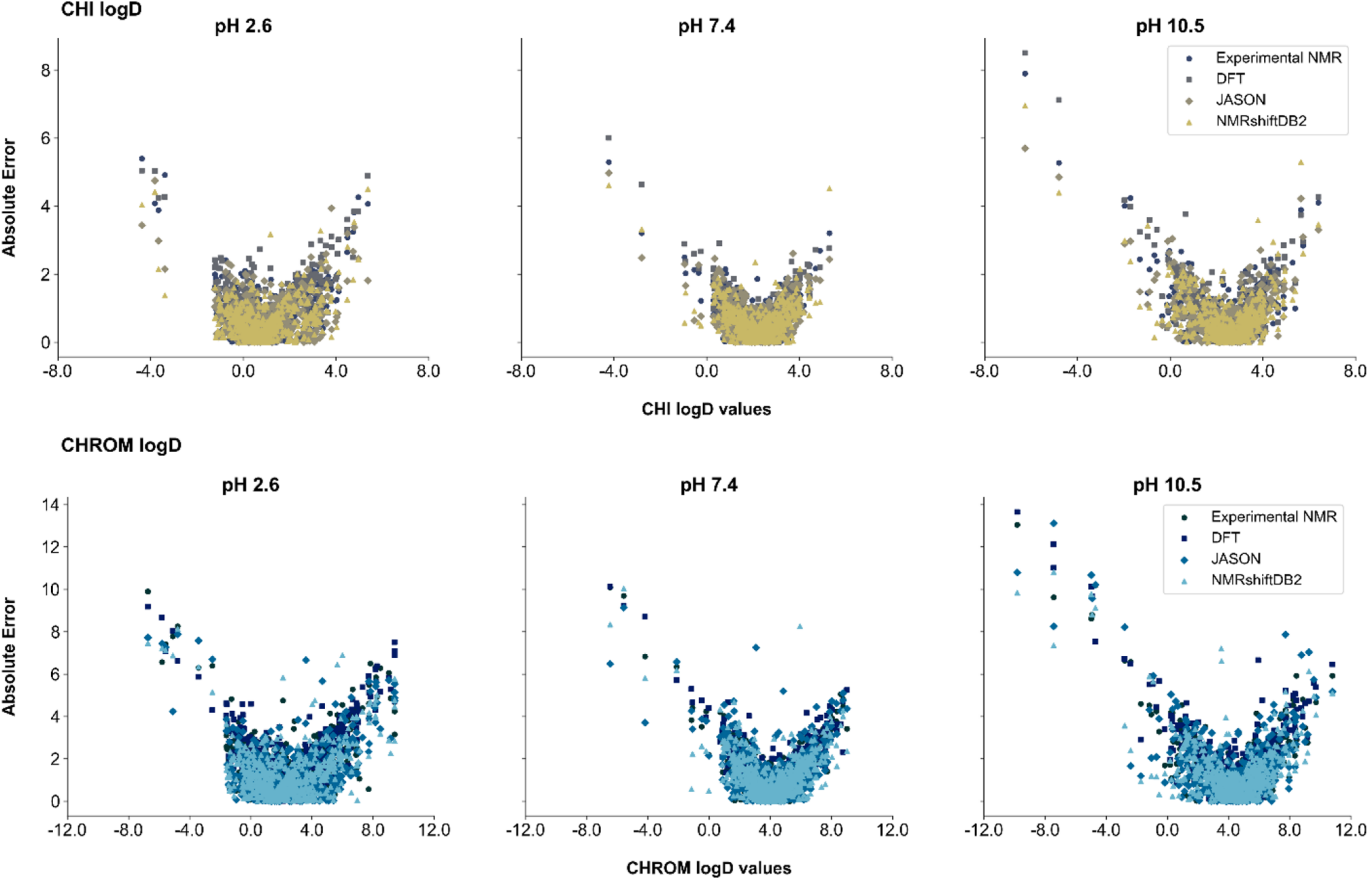
Distribution of the absolute prediction error for Gradient Boosting models as a function of the predicted parameter. The upper panel corresponds to the CHI logD parameter at three different pH values: 2.6, 7.4, and 10.5; whereas the lower panel represents the Chrom logD parameter at the same pH values. The data series indicates the source of input data (^1^H NMR spectra) used for training the machine learning models.

The chemical structures used to build the training and testing datasets for the ML models are identical to those described in the previous work.^15^ The distribution of outliers for each method of generating ^1^H NMR spectra relative to experimental spectra was analyzed under three distinct pH conditions for each logD parameter. As shown in Figure 5, which presents absolute prediction errors as a function of the respective logD parameter values, the outliers are gathered in two specific regions. The first region corresponds to negative logD values for both parameters across all three pH conditions. The second region is characterized by extremely high positive logD values. A key observation is that these two regions are consistent across all methods of computer-generated spectra as well as for experimental ^1^H NMR-based models. This suggests that machine learning models struggle to predict values near the boundaries of the available logD range due to the limited representation of compounds with such logD values. The training and testing datasets were constructed using compounds obtained through drug design processes as potential active agents, which are constrained to specific property ranges, including logD values. Consequently, the limited representation of boundary values in these datasets likely contributes to the model’s reduced prediction accuracy in these regions.

### Mixing Datasets

**Figure 6:**
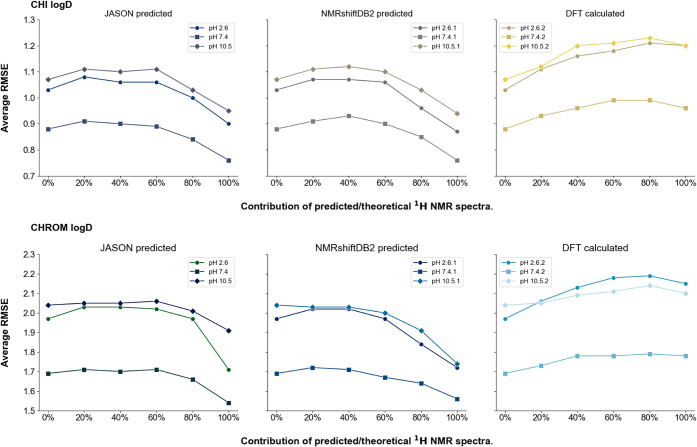
Panel presents the mean RMSE values obtained for the Gradient Boosting models using the 10CV method for mixed input datasets. Each graph illustrates the trend of mean RMSE values as the contribution of generated ^1^H NMR spectra of a given type increases within the set of experimental ^1^H NMR spectra. The upper panel displays data for the CHI logD parameter, while the lower panel corresponds to the Chrom logD parameter. The series within each panel represents the modeled parameter values at different pH levels.

Mixed ^1^H NMR spectral datasets (combining experimental and theoretical or predicted spectra in specific ratios) were used to build the input database for training predictive models. Randomly generated training sets were created, containing an increasing proportion of computer-generated spectra relative to experimental spectra. These sets were then used to train Gradient Boosting models employing the 10CV method (Figure 6).

In the first case, when the contribution of JASON- or NMRshiftDB2-based spectra increases, a slight rise (3.9% for CHI logD, and 1.2% for Chrom logD, on average) in RMSE is observed until the ratio reaches approximately 40%. At a 60% proportion, an insignificant decline in the RMSE values occurred (−1.2% for CHI logD, and −0.74% for Chrom logD), with the metrics converging toward levels characteristic of models based solely on predicted spectra. The change in RMSE error values for a set of 60% contribution of predicted spectra relative to experimental ^1^H NMR spectra to a set with 100% contribution of theoretical spectra for CHI logD models averaged −15.2% and −11.0% for Chrom logD. Ultimately, with the datasets entirely composed of predicted spectra, the lowest RMSE values are achieved, with RMSE values declining from a pure experimental set to a fully predicted ^1^H NMR spectra set by −13.0% for CHI logD and −10.6% for Chrom logD on average. In the second case, which examined the increasing contribution of DFT-based spectra, the RMSE gradually increased until the proportion of theoretical spectra reached 80%. The increase in RMSE values from a set represented only by experimental ^1^H NMR spectra to a set with 80% of theoretical spectra was 15.2% for CHI logD and 7.3% for Chrom logD, respectively. However, a slight decrease in RMSE values is noted at 100% theoretical spectra when compared to 80% theoretical dataset contribution (−2.10% for CHI and −0.5% for Chrom logD). Increasing the contribution of DFT data introduces factors that gradually degrade the predictive ability of the model, as the RMSE for the model trained exclusively on DFT data is higher in every case compared to the model based solely on experimental ^1^H NMR spectra. As a result, supplementing missing experimental spectra in the training set by using DFT proves ineffective and leads to poorer outcomes. Conversely, adding predicted spectra derived from JASON or NMRshiftDB2 allows for the supplementation of missing records in the spectral database, provided that a slight compromise in the model’s predictive power is acceptable. Despite the compatibility of data formats, chemical shift ranges, and identical feature vector structures, ^1^H NMR spectra and their generated counterparts are not fully compatible.

In summary, the best predictions are achieved by Gradient Boosting models trained on homogeneous datasets, regardless of whether they consist of purely experimental data or spectra derived from predictions.

### External Validation Sets

**Figure 7:**
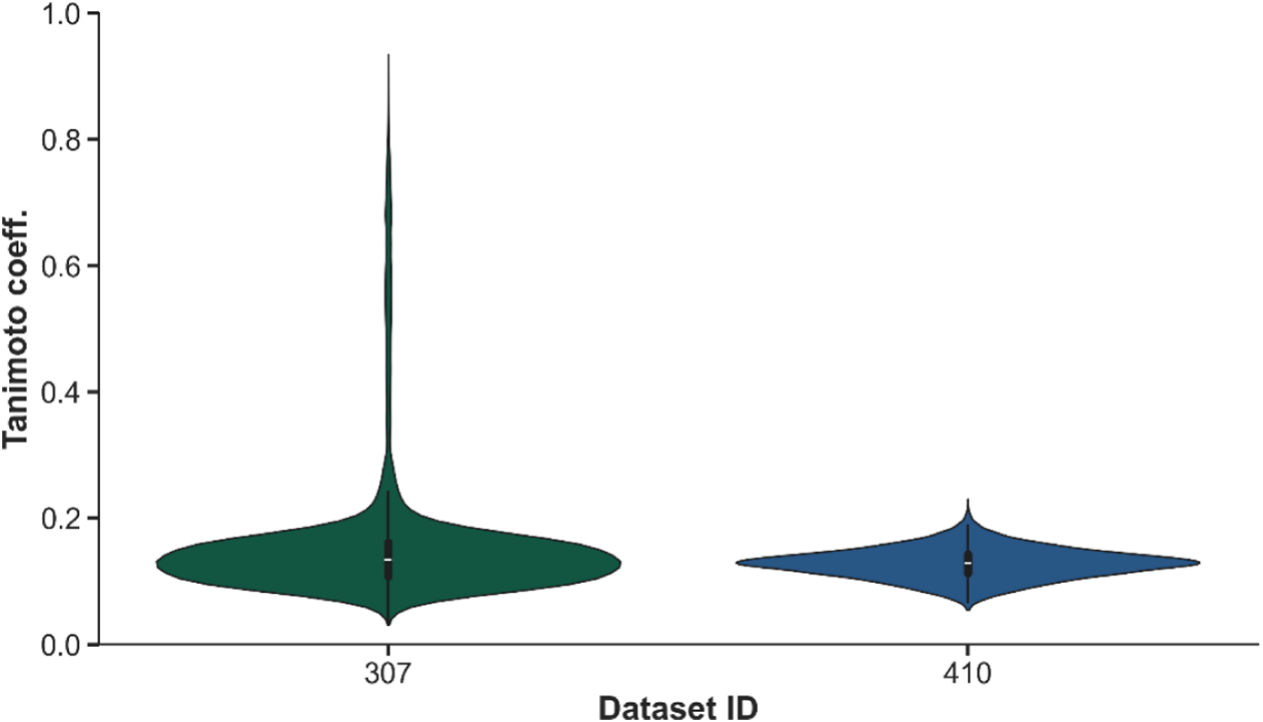
Violin plots showing the distribution of Tanimoto similarity coefficients (using ECFP4 fingerprints) between each compound in the external datasets (307 and 410) and all compounds in the training set.

The CHI logD and Chrom logD were measured for two additional sets of chemical compounds originating from separate projects, labeled as “dataset 307” and “dataset 410,” containing 65 and 11 compounds, respectively. ^1^H NMR spectra were recorded for all of these compounds. These compounds are characterized by unique structural motifs that were not present in the training set. To assess the similarity of external datasets to the training set, Tanimoto coefficients were calculated between each compound in the external datasets and every compound in the training set, using the ECFP4 fingerprint (Figure 7). Since ^1^H NMR focuses on atomic nuclei and their immediate chemical environments, the ECFP4 fingerprint was chosen for its comparable emphasis on capturing local structural information within a molecular radius.

**Figure 8:**
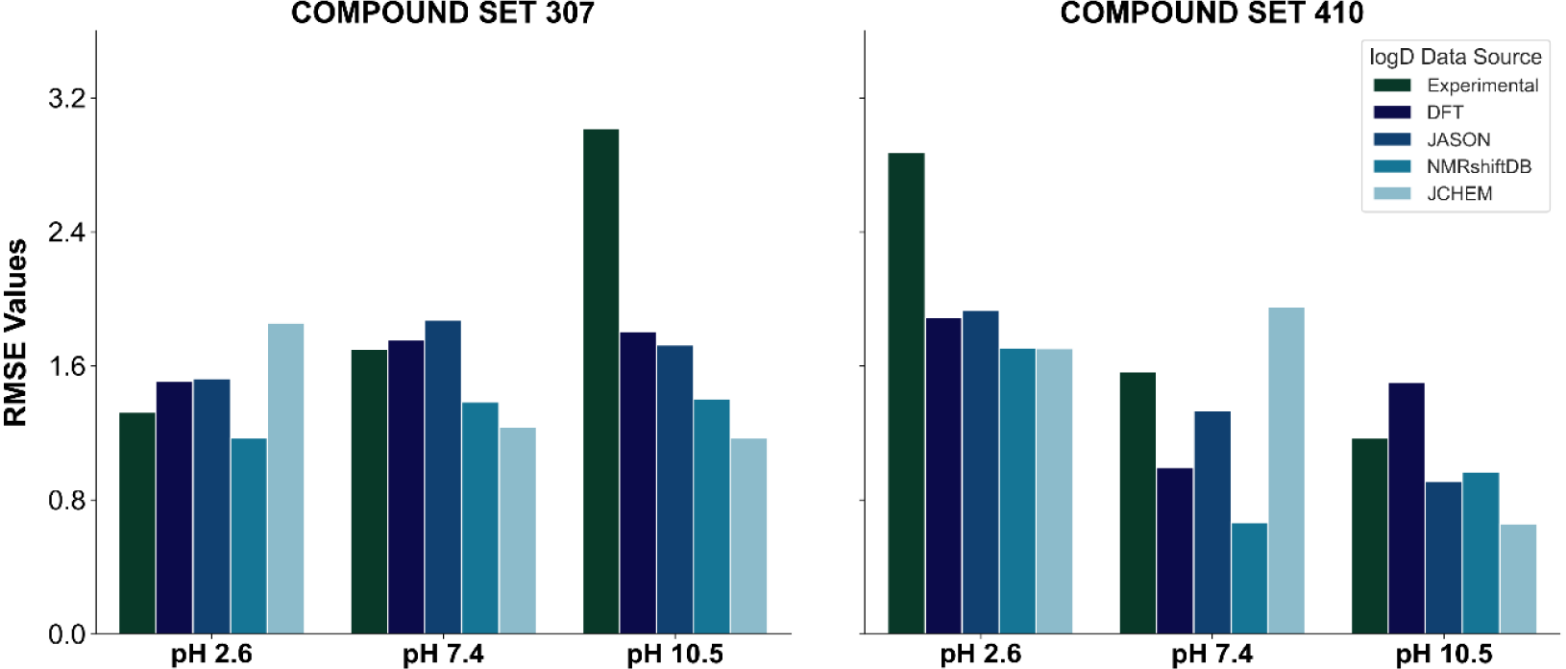
Bar charts present CHI logD parameter RMSE metrics for the validation query of the trained Gradient Boosting models for a given type of ^1^H NMR input data. The query was performed using two sets of ^1^H NMR spectra corresponding to structural motifs that were absent in the training and testing datasets. The last bar in each chart shows data obtained from logD value predictions using Instant JChem, as the CHI logD value can be directly compared with logD.

The results indicate low similarity for both datasets: the 307 dataset had a mean Tanimoto coefficient of 0.15 (SD = 0.10), while the 410 dataset showed a mean of 0.13 (SD = 0.02) (Figure 7). These low values suggest that the compounds in the external datasets have distinct structural features, showing minimal overlap with the training set. Both datasets were used to evaluate the Gradient Boosting models developed in this study. The results obtained using these models are presented in Figure 8.

The average RMSE values obtained for dataset 307 in the context of CHI logD model predictions under varying pH conditions exhibit values of 1.38, 1.68, and 12.24 for pH values of 2.6, 7.4, and 10.5, respectively. However, it is important to note that the RMSE obtained based on experimental data for pH 10.5 was as high as 3.02. The best results were achieved for models trained on data generated using the NMRshiftDB2 predictor, where average RMSE values were 1.17, 1.39, and 1.40 for increasing pH values. In comparison, models based on data from the commercial logD predictor implemented in Instant JChem software exhibited RMSE values of 1.86, 1.23, and 1.17, respectively. The significantly high RMSE value of 3.02 for models based on experimental ^1^H NMR spectra at pH 10.5 may indicate potential measurement errors in the CHI logD values at this pH for certain compounds. For dataset 410 CHI logD, models trained on data from the NMRshiftDB2 predictor also achieved the best results, with average RMSE values of 1.71, 0.67, and 0.97 for increasing pH values, compared to models based on JChem data, which obtained RMSE values of 1.70, 1.95, and 0.66, respectively. Again, an outlying RMSE value of 2.88 at pH 2.6 for one model based on experimental spectra suggests potential data quality issues under specific pH conditions. Apart from these anomalies, there is no clear trend regarding the impact of the spectral generation method on the obtained RMSE values. For Chrom logD predictions, no comparative data are available for the JChem, limiting the analysis to models trained on different datasets. The RMSE values for the NMRshiftDB2 predictor in dataset 307 were 1.47 and 2.24 for pH 2.6 and 7.4, respectively, while for dataset 410, they were 1.71 and 1.13. It should be emphasized that two cases (data set 307: RMSE 2.55 and dataset 410: RMSE = 1.97) at pH 10.5 showed significantly higher RMSE values. Overall, models based on NMRshiftDB2 data achieved the lowest RMSE values; however, no clear trends were observed regarding differences in results depending on the pH conditions and validation sets for other spectrum generation methods.

To further assess the statistical significance of the differences between logD prediction methods, pairwise *t*-tests were conducted on the absolute error (MAE) distributions for both datasets. In the case of data set 307, the majority of method comparisons yielded statistically significant differences (*p* < 0.05), particularly between experimental data and computational predictors. Conversely, for dataset 410, the results were more balanced, with some method comparisons exhibiting significant differences while others did not. These findings suggest that while systematic deviations exist between methods, their impact may be dataset-dependent. Detailed statistical results and corresponding box plots are provided in Figures S4–S7.

### Normalized RMSE

Although the RMSE is a widely used metric for evaluating the performance of regression models, it has its limitations. For this reason, when models using RMSE are compared, caution must be exercised, and the characteristics of this metric must be taken into account. As shown in eq 1, the RMSE value depends on the range of the data on which the model operates. In practice, this means that if one model predicts values in the range of (0, 1000), while another in the range of (0, 10), direct comparison of their RMSE values may lead to erroneous conclusions, even if the relative predictive quality of the models is similar. Therefore, comparing ML models trained on datasets with different input data ranges and characteristics is methodologically incorrect. However, to select optimal training datasets or compare their models to those reported in the literature, relative comparisons of results are often necessary. To facilitate such comparisons, several RMSE normalization methods have been described in the literature,^61−64^ with the most commonly used ones presented in eqs 2–4.
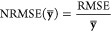
2
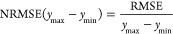
3
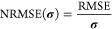
4

**Figure 9:**
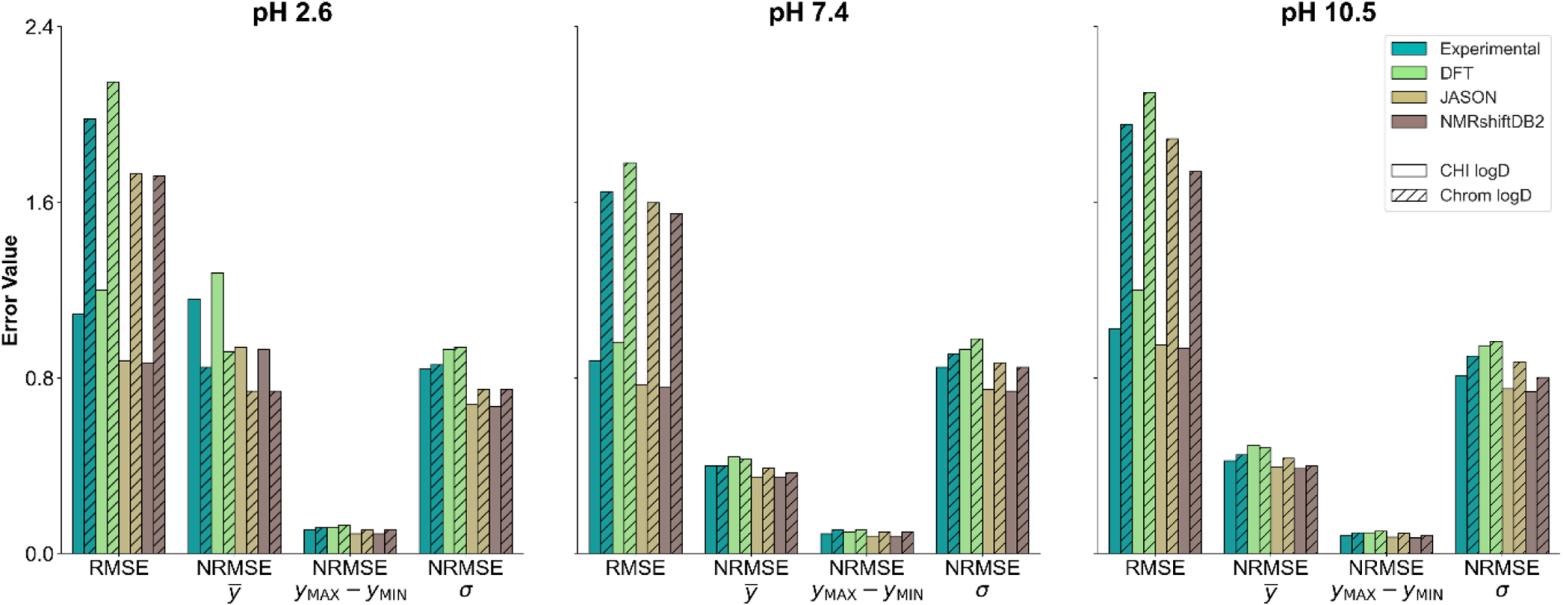
Bar plots illustrate the performance metrics (RMSE and NRMSE) for Gradient Boosting models trained to predict CHI logD and Chrom logD at three different pH levels (2.6, 7.4, and 10.5). Each pair of bars corresponds to CHI logD (plain bars) and Chrom logD (hatched bars) for each of the four input data sources: Experimental ^1^H NMR spectra were obtained using three spectral generation methods (DFT, JASON, and NMRshiftDB2). The metrics are displayed in four categories: RMSE, NRMSE scaled by the mean of the target values (**y̅**), NRMSE scaled by the range of the target values (*y*_**max**_**–***y*_**min**_), and NRMSE scaled by the standard deviation of the target values (σ).

Normalized metrics enable the evaluation of model prediction quality by taking into account the average values in the dataset (eq 2), comparisons relative to the range of data values (eq 3), or estimation of model error relative to the standard deviation in the dataset (eq 4). Table 1 presents the values of all three statistical metrics for the CHI logD and Chrom logD datasets across the full pH range. It is noteworthy that the average values, value ranges, and standard deviations in the CHI logD datasets are approximately 50%–60% of the average values for the Chrom logD datasets, which corresponds to similar trends observed when comparing RMSE values for these systems (Figure 9).

**Table 1:** Statistical Metrics of Datasets for the CHI and Chrom LogD at Different pH Levels

	pH 2.6		pH 7.4		pH 10.5	
logD parameter	CHI	Chrom	CHI	Chrom	CHI	Chrom
***y̅***^a^	0.94	2.33	2.21	4.14	2.42	4.34
*y*_**max**_**–***y*_**min**_	9.74	16.14	9.52	15.50	12.67	20.64
**σ**	1.29	2.29	1.03	1.82	1.27	2.17

a In this table, **y̅** represents the mean value of logD labels used in the datasets, *y*_max_–*y*_min_ indicates the range, i.e., the difference between the maximum and minimum actual values of the predicted parameter, and σ denotes the standard deviation of the actual parameter values.

In the presented study, the models were trained based on CHI logD and Chrom logD. Although both parameters exhibit a linear relationship,^65^ the Chrom logD datasets contained, on average, 100 more compounds. Nevertheless, the RMSE values obtained for models based on Chrom logD were approximately twice as high as those for CHI logD. It is unlikely that advanced ML algorithms would fail to account for this linear relationship, which suggests that other factors may contribute to the significantly higher RMSE values. Only by comparing the RMSE values with the normalized values (Figure 9) is it revealed that the models based on both parameters demonstrate comparable predictive capability. Significant differences (on average, 0.27 units of RMSE) are observed only for NRMSE(**y̅**) at pH 2.6, which is likely due to a high number of outliers and the fact that the mean does not reflect the true central value of the dataset. For the remaining metrics, these differences are insignificant for data at a given pH and do not indicate substantial differences between models based on the CHI logD and Chrom logD.

The analysis of normalized metric values (Figure 9) indicates that despite the 2-fold difference in RMSE values, the models exhibit similar predictive performance. This can be attributed to differences in the characteristics of the datasets, indicating that the comparison of RMSE for models trained on datasets with differing value ranges is inappropriate. Our results suggest that RMSE should only be used to compare models that are trained and tested on datasets with similar statistical properties, such as mean, distribution, or standard deviation—even if these datasets are not identical. In other cases, where the goal is to compare the predictive abilities of models, normalized metrics such as NRMSE should be applied.

## Conclusions

This study builds on our prior research, where we developed a novel descriptor based on experimental ^1^H NMR spectra as the core input for ML models to predict logD. The initial application of experimental NMR spectra as the foundation for our descriptor demonstrated predictive performance comparable to that of traditional molecular descriptors. However, practical challenges, such as solvent effects and instrumental variability, motivated the transition to theoretical spectra, which provided a more controlled and reproducible input while maintaining predictive accuracy. To address these challenges, we investigated the use of predicted and theoretical ^1^H NMR spectra to eliminate the dependency on experimental measurements, creating an entirely computational workflow that could streamline ML-based logD prediction without experimental constraints.

Our findings reveal that theoretical ^1^H NMR spectra—particularly those generated by NMRshiftDB2 and JEOL JASON—can rival and even surpass experimental spectra in machine learning (ML) applications for logD prediction. This shift toward theoretical spectra offers clear advantages: it eliminates the need for labor-intensive experimental pipelines, bypasses challenges related to solubility, sample purity, and data inconsistencies, and provides a scalable, universally applicable approach that does not rely on specialized laboratory resources.

A key insight from our study is that only two methods—JASON and NMRshiftDB2—outperformed experimental spectra. Their success is likely attributed to their foundation: rather than generating purely theoretical spectra, these models are trained on tens of thousands of real experimental spectra. As a result, they inherently capture complex spectral relationships that are naturally present in empirical data, bridging the gap between the raw experimental measurements and computational predictions.

Moreover, our approach treats NMR spectra as holistic fingerprints rather than dissecting individual peak positions or spin–spin interactions. This representation aligns well with ML methodologies, where capturing overarching spectral patterns proves to be more effective than analyzing fine structural details. While DFT-based spectra remain a gold standard in quantum mechanical analyses, their computational intensity and unexpectedly lower predictive power in our study suggest that they are less suited for large-scale applications, such as logD prediction. In contrast, HOSE-code-based methods and ML-driven spectral models demonstrate a more efficient and robust alternative.

Another crucial takeaway is the impact of data consistency. Mixing experimental and predicted spectra resulted in diminished performance compared to using homogeneous datasets, reinforcing the importance of maintaining uniformity in spectral inputs. Finally, our results highlight the necessity of normalized error metrics, such as NRMSE, for ensuring reliable model comparisons across datasets with varying numerical scales.

Together, these insights not only refine our understanding of spectral representations in ML but also pave the way for more accessible, scalable, and data-driven approaches to molecular property prediction. Validation against external datasets further demonstrated the robustness and transferability of our models, as they performed on par with established commercial software like JChem. This comparison underscores the reliability and applicability of our computational pipeline, confirming that the predicted ^1^H NMR spectra can serve as an effective alternative to experimental data for logD predictions. By achieving comparable accuracy to industry-standard tools without reliance on costly and time-intensive experimental procedures, our approach offers a scalable, accessible solution that holds promise for widespread adoption in cheminformatics and drug discovery workflows. This methodology, by replacing experimental input entirely, opens new possibilities for ML in chemical property prediction and remains adaptable for future advancements in computational chemistry and cheminformatics.

## Data Availability

All scripts and input data, including both theoretical and experimental ^1^H NMR spectra formatted as ready-to-use inputs for machine learning models, are available in the GitHub repository at https://github.com/Prospero1988/NMR-AI_part2. The entire repository is provided under the MIT License.

## Supporting Information Available

Detailed distribution of RMSE values across various computational sets (functional + basis sets) used in the comparative benchmarking of DFT methods for accurate ^1^H NMR predictions, along with corresponding computation times (PDF)

## Abbreviations

10CV: 10-fold cross-validation
^1^H NMR: Proton nuclear magnetic resonance
6-31G(2d): Split-valence double-ζ basis set with polarization functions
6-311+G(2d,p): Triple-zeta valence basis set with diffuse and polarization functions
AdaBoost: Adaptive boosting
AI: Artificial Intelligence
B3LYP: Becke, 3-parameter, Lee–Yang–Parr hybrid functional
CAM-B3LYP: Coulomb-attenuating method with B3LYP functional
CDCl_3_: Deuterated chloroform
CDK: Chemistry Development Kit
CHI logD: Chromatographic hydrophobicity index logD
Chrom logD: Chromatographically determined octanol–water partition coefficient
cc-pVDZ: Correlation-consistent polarized valence double-zeta basis set
cc-pVTZ: Correlation-consistent polarized valence triple-zeta basis set
def2-SVP: Karlsruhe split-valence polarized basis set
def2-TZVP: Karlsruhe triple-ζ valence polarized basis set
D3: Grimme’s dispersion correction
D3BJ: Grimme’s dispersion correction with Becke–Johnson damping
DFT: Density functional theory
DMSO-*d*_6_: Deuterated dimethyl sulfoxide
ECFP: Extended-connectivity fingerprints
FID: Free induction decay
GenECP: General effective core potentials for Gaussian calculations
GIAO: Gauge-independent atomic orbital computational method
Gradient Boosting: An ensemble ML method that combines weak learners to improve model performance
HOSE: Hierarchical organization of spherical environment codes
HPLC: High-performance liquid chromatography
JEOL JASON: Software used for NMR spectra prediction
LightGBM: Light gradient boosting machine
logD: Logarithm of the distribution constant
LR: Logistic regression
MACCS: Molecular ACCess System fingerprints
ML: Machine learning
M06-2X: Minnesota 06 meta-hybrid functional with double exchange
NMR: Nuclear magnetic resonance
NMRshiftDB2: Nuclear magnetic resonance shift database
PBEPBE: Perdew–Burke–Ernzerhof functional within generalized gradient approximation
PCM: Polarizable continuum model
RDKit: Open-source toolkit for cheminformatics
RF: Random forest
RMSE: Root mean squared error
SD: Standard deviation
SMILES: Simplified molecular input line entry system
SVM: Support vector machines
SVR: Support vector regression
TMS: Tetramethylsilane
ωB97XD: Range-separated hybrid functional with empirical dispersion
XGBoost: Extreme gradient boosting
