# Supplementary material for: From NMR to AI: Do We Need 1H NMR Experimental Spectra to Obtain High-Quality logD Prediction Models?
Source: J Chem Inf Model. 2025 Mar 5;65(6):2924–39. doi: 10.1021/acs.jcim.4c02145 (PMC11938277; doi:10.1021/acs.jcim.4c02145)
Supplement: Supplementary file 1 — ci4c02145_si_001.pdf [file ci4c02145_si_001.pdf]

From NMR to AI: Do we need ^{1}H NMR experimental spectra to obtain high-quality logD prediction models?

Arkadiusz Leniak,[1] Wojciech Pietruś,[2] Aleksandra Świderska,[1] Rafał Kurczab[3*]*

[1] Department of Medicinal Chemistry, Celon Pharma S.A., ul. Marymoncka 15, 05-152 Kazuń Nowy, Poland

[2] Department of Medicinal Chemistry, Maj Institute of Pharmacology, Polish Academy of Sciences, Smetna 12, 31-343 Krakow, Poland.

[3] University of Applied Sciences in Tarnow, Faculty of Mathematics and Natural Sciences, Department of Chemistry, Mickiewicza 8, 33-100 Tarnow, Poland

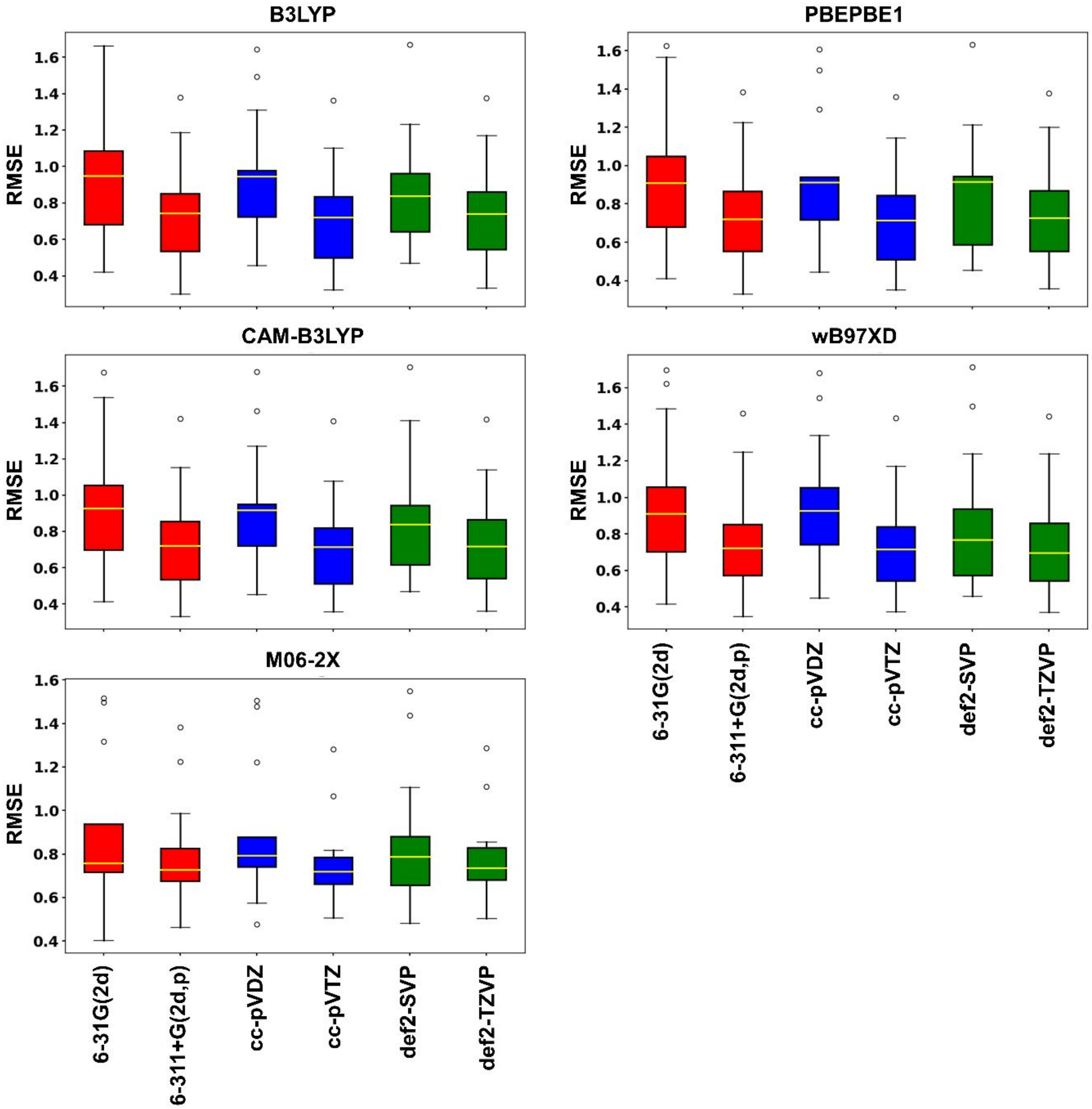

Figure S1. Boxplots showing the RMSE values for each functional and its associated basis sets, comparing their predictive accuracy in the **DMSO environment**. Each box represents the distribution of RMSE values across different basis sets for a given functional, illustrating the variability in performance within each computational set. Lower and more compact RMSE distributions indicate better and more consistent performance across basis sets.

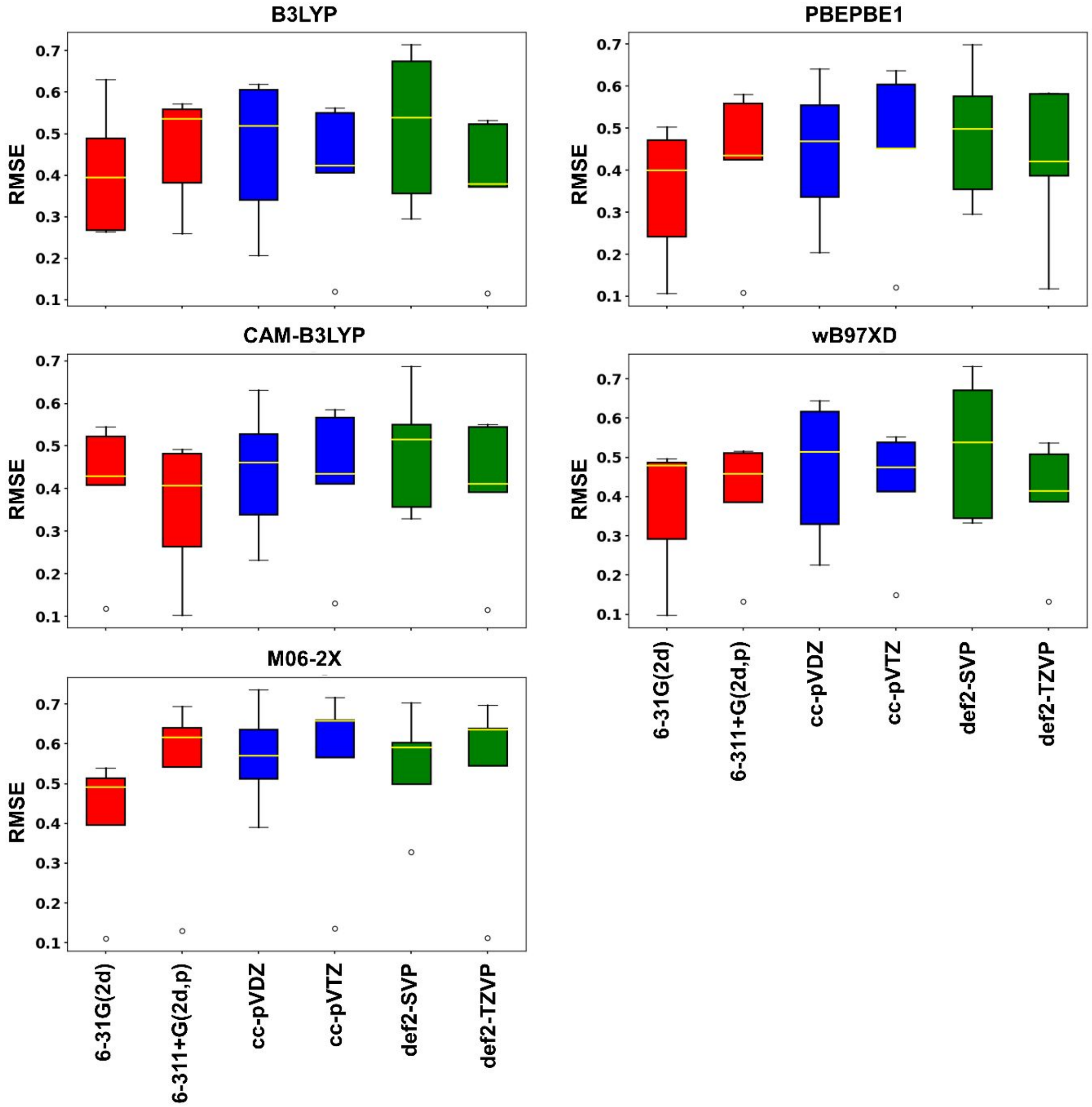

Figure S2. Boxplots showing the RMSE values for each functional and its associated basis sets, comparing their predictive accuracy in the $CDCl_3$ environment. Each box represents the distribution of RMSE values across different basis sets for a given functional, illustrating the variability in performance within each computational set. Lower and more compact RMSE distributions indicate better and more consistent performance across basis sets.

Table S1: Benchmarking of computational sets across DMSO and $CDCl_3$ solvents, ranked by mean RMSE values. The table lists each functional-basis set combination with its respective mean RMSE, standard deviation (Std RMSE), and rank in DMSO and $CDCl_3$. The overall performance ranking (SUM RANK) was determined by summing the individual rankings from each solvent. Lower RMSE and higher consistency across solvents indicate better-performing computational sets.

Computation Set	DMSO			$CDCl_3$			SUM RANK
	Mean RMSE	*Std RMSE*	*Rank*	*Mean RMSE*	*Std RMSE*	*Rank*	
CAM-B3LYP + 6-311+G(2d,p)	0.74	0.30	5	0.35	0.17	2	1
B3LYP + def2-TZVP	0.74	0.30	6	0.38	0.17	4	2
B3LYP + cc-pVTZ	0.72	0.29	1	0.41	0.18	11	3
wB97XD + def2-TZVP	0.75	0.32	9	0.40	0.16	5	4
CAM-B3LYP + def2-TZVP	0.75	0.29	8	0.40	0.18	7	5
CAM-B3LYP + cc-pVTZ	0.72	0.29	2	0.43	0.18	15	6
wB97XD + 6-311+G(2d,p)	0.77	0.32	12	0.40	0.16	6	7
PBEPBE + cc-pVTZ	0.73	0.29	3	0.45	0.20	18	8
wB97XD + cc-pVTZ	0.74	0.30	7	0.43	0.16	14	8
PBEPBE + def2-TZVP	0.75	0.30	10	0.42	0.19	12	10
B3LYP + 6-311+G(2d,p)	0.74	0.31	4	0.46	0.14	20	11
PBEPBE + 6-311+G(2d,p)	0.76	0.31	11	0.42	0.19	13	11
PBEPBE + 6-31G(2d)	0.93	0.40	26	0.34	0.17	1	13
M062X + 6-31G(2d)	0.90	0.35	21	0.41	0.18	10	14
wB97XD + 6-31G(2d)	0.96	0.41	30	0.37	0.17	3	15

CAM-B3LYP + 6-31G(2d)	0.94	0.39	27	0.41	0.17	8	16
B3LYP + 6-31G(2d)	0.95	0.40	29	0.41	0.16	9	17
PBEPBE + def2-SVP	0.88	0.33	17	0.49	0.16	22	18
PBEPBE + cc-pVDZ	0.91	0.37	23	0.44	0.17	17	19
CAM-B3LYP + cc-pVDZ	0.92	0.36	24	0.44	0.16	16	19
M062X + def2-TZVP	0.78	0.21	14	0.53	0.24	27	21
M062X + 6-311+G(2d,p)	0.81	0.25	15	0.53	0.23	26	21
M062X + cc-pVTZ	0.77	0.20	13	0.55	0.24	29	23
wB97XD + def2-SVP	0.89	0.38	18	0.52	0.18	25	24
M062X + def2-SVP	0.87	0.32	16	0.55	0.14	28	25
B3LYP + def2-SVP	0.89	0.33	20	0.52	0.19	24	25
B3LYP + cc-pVDZ	0.93	0.37	25	0.46	0.18	19	25
CAM-B3LYP + def2-SVP	0.90	0.36	22	0.49	0.15	23	28
M062X + cc-pVDZ	0.89	0.32	19	0.57	0.13	30	29
wB97XD + cc-pVDZ	0.94	0.38	28	0.47	0.18	21	29

Table S2: Comparison of computation times for geometry optimization and ^{1}H NMR calculations across selected compounds, using CAM-B3LYP/6-311+G(2d,p) versus B3LYP/def2-TZVP and B3LYP/cc-pVTZ. Negative values (marked in red) indicate longer computation times for CAM-B3LYP/6-311+G(2d,p) relative to the compared set. Optimization and ^{1}H NMR calculation times were benchmarked to assess the efficiency of each computational set in terms of speed and resource usage.

Compound	Optimization time (*CAM-B3LYP/6-311+G(2d,p)* vs *B3LYP/def2-TZVP*)	Optimization time (*CAM-B3LYP/6-311+G(2d,p)* vs *B3LYP/cc-pVTZ*)	NMR calc. time (*CAM-B3LYP/6-311+G(2d,p)* vs *B3LYP/def2-TZVP*)	NMR calc. time (*CAM-B3LYP/6-311+G(2d,p)* vs *B3LYP/cc-pVTZ*)
302-345-02	–01:20:32	02:08:18	00:06:27	00:09:25
302-495-01	–00:36:51	00:38:28	00:04:52	00:11:05
302-566-01	–00:15:33	01:28:15	00:05:49	00:09:36
302-577-01	–01:05:21	00:34:51	00:08:14	00:11:03
305-712-02	–01:50:06	01:18:01	00:07:22	00:09:04
305-757-02	–00:25:09	07:28:27	00:05:40	00:10:22
306-044-02	–00:03:24	02:39:23	00:06:13	00:10:31
307-209-04	00:35:01	03:37:22	00:07:05	00:11:53
307-503-06	–00:09:43	01:25:48	00:04:19	00:08:26
504-228-01	–00:46:11	00:06:10	00:02:13	00:04:28
600-158-01	–00:16:37	06:05:01	00:08:23	00:15:17
600-181-01	–00:12:44	00:30:50	00:00:49	00:01:44
601-500-03	–00:04:37	02:18:24	00:03:13	00:05:14
601-506-01	00:03:14	01:17:07	00:01:37	00:03:12
601-803-01	–04:29:55	02:58:39	00:46:17	00:46:39
307-013-03	–03:07:57	01:04:10	00:05:47	00:08:27
600-126-01	00:52:43	01:31:58	00:04:26	00:03:35
600-158-01	01:47:59	03:49:34	00:08:57	00:17:24
601-804-03	09:45:26	01:01:18	00:08:53	00:10:42

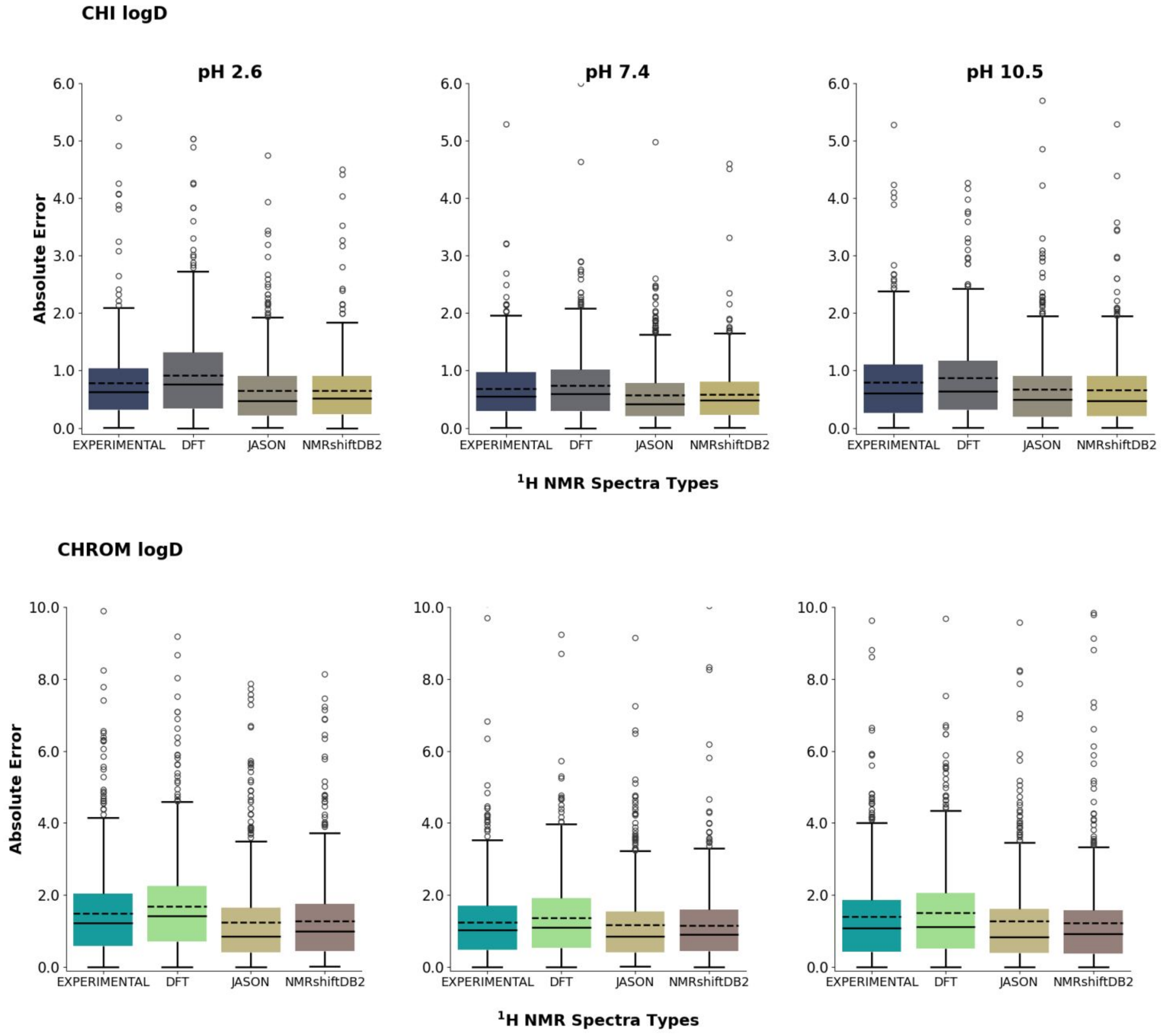

Figure S3. An expanded version of Figure 4 from the main manuscript. The diagrams additionally indicate the outlier points for each boxplot. Boxplots of Absolute Errors between computer-generated and experimental predictions of CHI logD (top) and Chrom logD (bottom) at different pH. In each plot, the first box corresponds to experimental ^{1}H NMR spectra, whereas the remaining ones are the methods used to obtain the generated ^{1}H NMR spectra. In each box, the horizontal dashed line describes the mean value, while the solid line refers to the median.

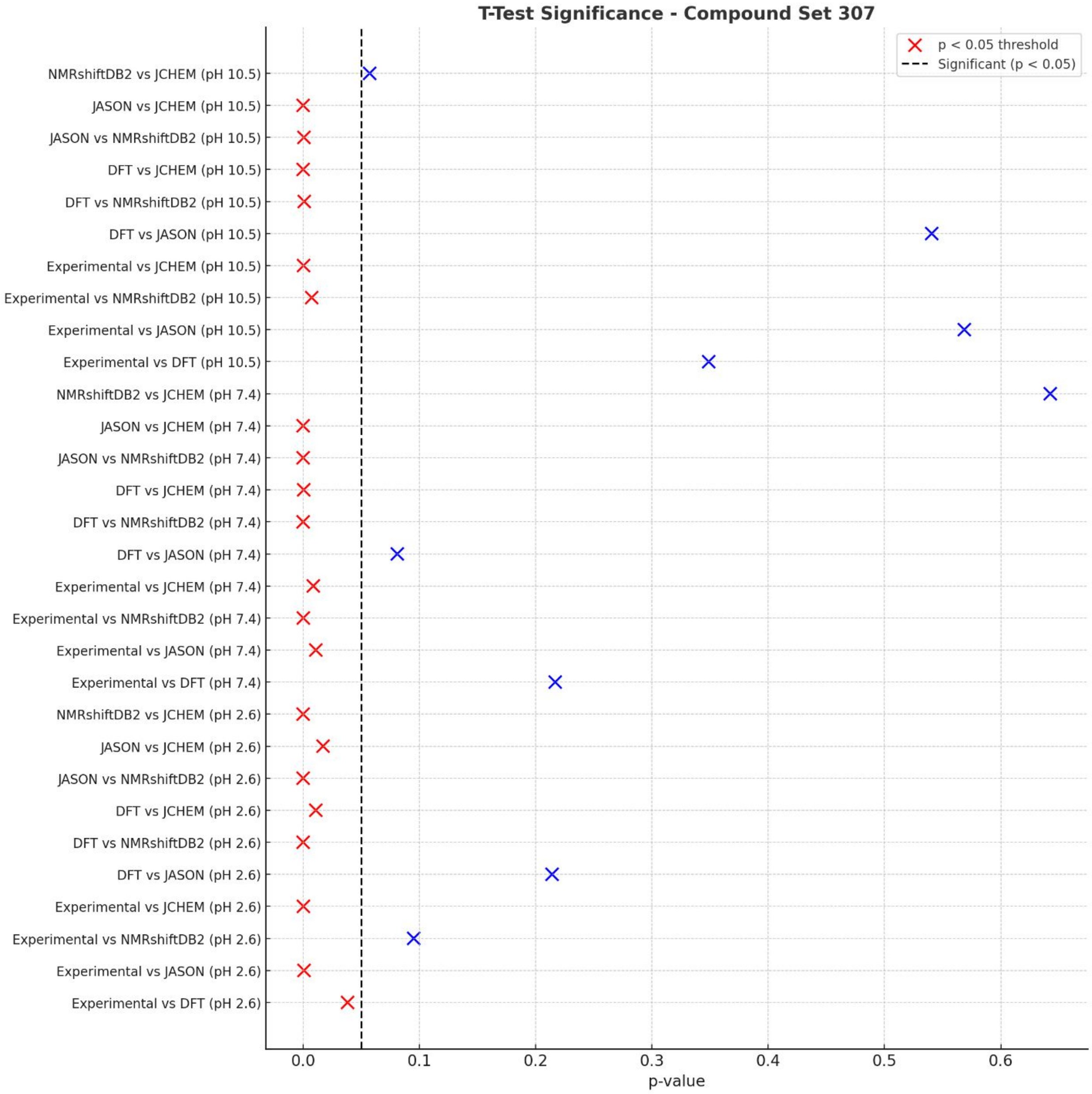

Figure S4. Statistical significance of pairwise t-tests for Compound Set 307. This figure presents the results of pairwise t-tests comparing the performance of different methods across three pH levels. The red markers indicate statistically significant differences ($p < 0.05$), while the blue markers denote non-significant differences ($p \geq 0.05$). The dashed vertical line

represents the conventional significance threshold of p = 0.05. Each comparison involves two methods evaluated under the same pH condition.

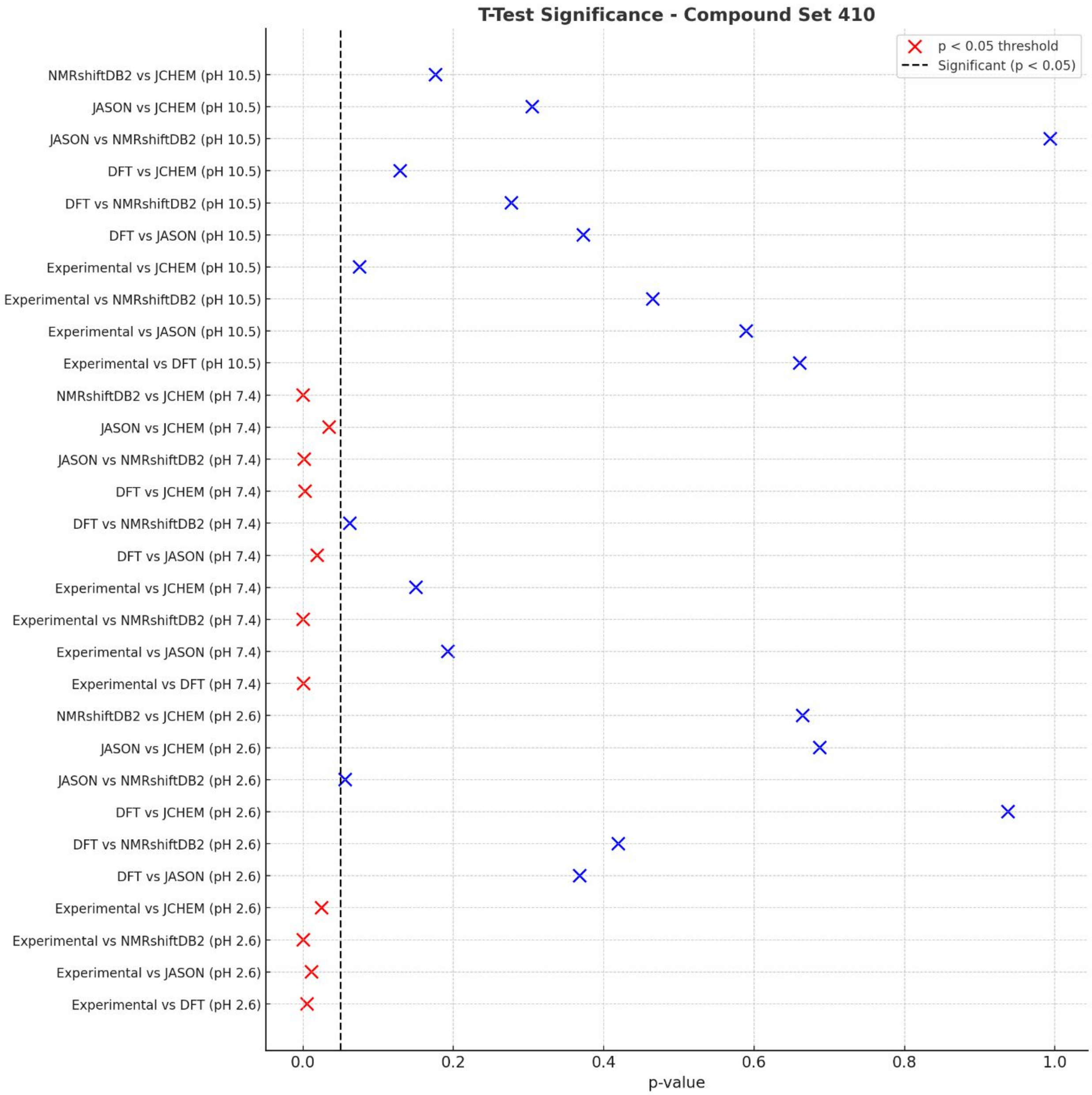

Figure S5. Statistical significance of pairwise t-tests for Compound Set 410.

This figure shows the statistical significance of pairwise t-tests conducted for Compound Set 410. The methodology follows the same approach as in Figure X, where red markers highlight

statistically significant differences ($p < 0.05$) and blue markers represent non-significant differences ($p \geq 0.05$). The dashed vertical line serves as a reference for the 0.05 significance level.

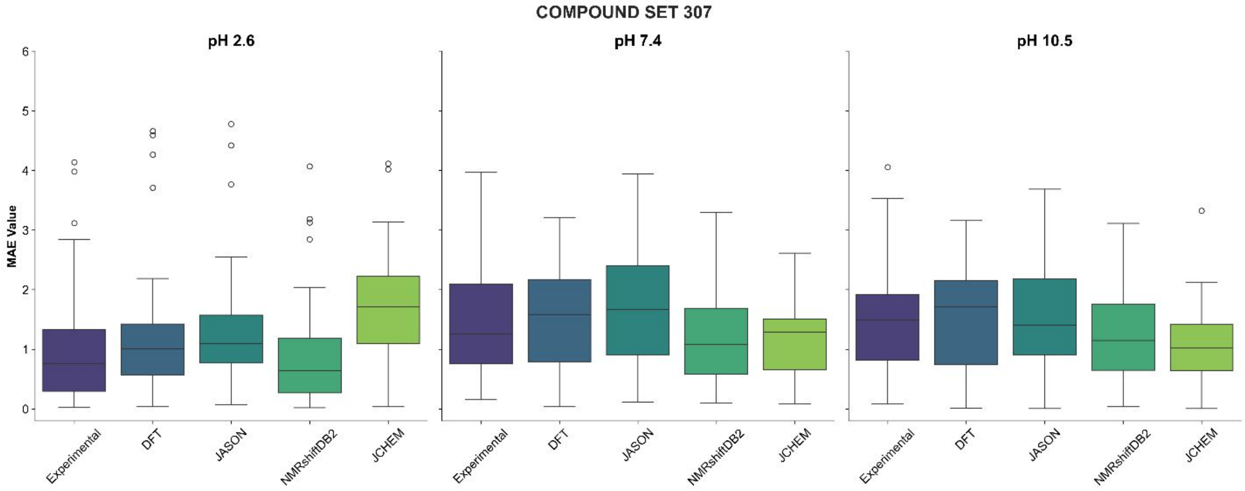

Figure S6. Boxplots of MAE values for different logD prediction methods in Compound Set 307. This figure presents boxplots of Mean Absolute Error (MAE) values for various logD prediction methods evaluated at three different pH levels (2.6, 7.4, and 10.5) in Compound Set 307. Each method's distribution is visualized, highlighting variability and potential outliers. The Experimental method serves as a reference, while computational methods (DFT, JASON, NMRshiftDB2, and JCHEM) are compared. Boxplots provide insights into method-specific accuracy and robustness across different pH conditions.

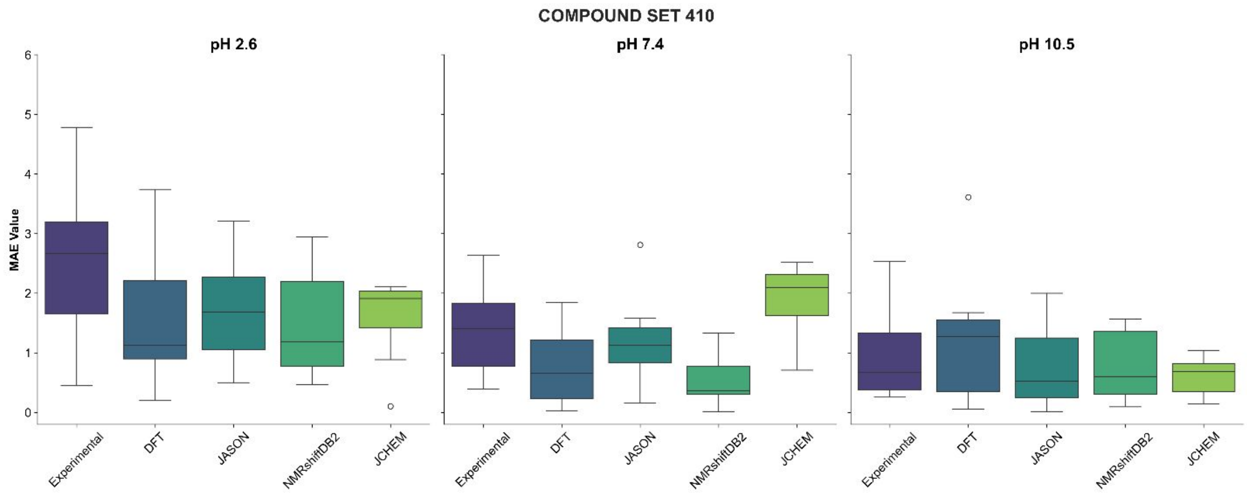

Figure S7. Boxplots of MAE values for different logD prediction methods in Compound Set 410. This figure illustrates the distribution of Mean Absolute Error (MAE) values for logD prediction methods applied to Compound Set 410 at three pH levels. The boxplots visualize median values, interquartile ranges, and outliers, allowing a direct comparison between experimental and computational approaches. Consistent trends and variability between methods indicate the relative performance of each prediction approach under different chemical conditions.
